# Relative Protein Intake and Physical Function in Older Adults: A Systematic Review and Meta-Analysis of Observational Studies

**DOI:** 10.3390/nu10091330

**Published:** 2018-09-19

**Authors:** Hélio José Coelho-Júnior, Luiz Milano-Teixeira, Bruno Rodrigues, Reury Bacurau, Emanuele Marzetti, Marco Uchida

**Affiliations:** 1 Applied Kinesiology Laboratory–AKL, School of Physical Education, University of Campinas, Av. Érico Veríssimo, 701, Cidade Universitária “Zeferino Vaz”, Barão Geraldo, Campinas-SP 13.083-851, Brazil; teixeira.luisfelipe@gmail.com (L.M.-T.); prof.brodrigues@gmail.com (B.R.); uchida@g.unicamp.br (M.U.); 2 Department of Geriatrics, Neurosciences and Orthopedics, Teaching Hospital “Agostino Gemelli”, Catholic University of the Sacred Heart, 00168 Rome, Italy; emarzetti@live.com; 3 School of Arts, Sciences and Humanities, University of São Paulo, Rua Arlindo Béttio, 1000-Ermelino Matarazzo, São Paulo-SP 03828-000, Brazil; reurybacurau@usp.br

**Keywords:** sarcopenia, protein intake, physical function

## Abstract

(1) Background: The present work aims to conduct a systematic review and meta-analysis of observational studies, in order to investigate the association of relative protein intake and physical function in older adults; (2) Methods: Observational studies, that investigated the association between protein intake and physical function in older adults, were retrieved from MEDLINE, SCOPUS, CINAHL, AgeLine, EMBASE, and Cochrane-CENTRAL. Two independent researchers conducted study selection and data extraction; (3) Results: Very high protein intake (≥1.2 g/kg/day) and high protein intake (≥1.0 g/kg/day) groups showed better lower limb physical functioning and walking speed (WS) performance, respectively, in comparison to individuals who present relative low protein (<0.80 g/kg/day) intake. On the other hand, relative high protein intake does not seem to propitiate a better performance on isometric handgrip (IHG) and chair rise in comparison to relative low protein intake. In addition, there were no significant differences in the physical functioning of high and middle protein intake groups; (4) Conclusions: In conclusion, findings of the present study indicate that a very high (≥1.2 g/kg/day) and high protein intake (≥1.0 g/kg/day) are associated with better lower-limb physical performance, when compared to low protein (<0.80 g/kg/day) intake, in community-dwelling older adults. These findings act as additional evidence regarding the potential need to increase protein guidelines to above the current recommendations. However, large randomized clinical trials are needed to confirm the addictive effects of high-protein diets (≥1.0 g/kg/day) in comparison to the current recommendations on physical functioning. All data are available in the Open ScienceFramework.

## 1. Introduction

Sarcopenia is a geriatric condition characterized by progressive muscle atrophy accompanied by loss of muscle strength and/or function [1]. The incidence of sarcopenia rises with aging and its prevalence is markedly increased in older subjects [2]. In the absence of targeted interventions, the clinical course of sarcopenia is marked by higher odds of mobility disability, loss of independence, and mortality [3,4,5,6]. In this sense, adequate protein intake and physical exercise have been suggested as the two main strategies to counteract sarcopenia, and prevent its deleterious effects [7,8].

Although protein supplementation may be advisable in the management of sarcopenia, the optimal protein requirement for older adults is presently unclear. Indeed, the established guidelines recommended for a number of agencies, such as the Dietary Allowance (RDA), RDI (recommended daily intake) [9], and the RNI (reference nutrient intake) [10] have been questioned, and researchers have discussed if the recommended protein intake is enough to maintain the functional status or even prevent its decline and muscle atrophy in older adults [11,12]. Most critical are regarding the RDA, so that the main concern is that the amount of protein recommended is based on nitrogen balance studies, which may be associated with a methodological bias [11,13].

Opinion articles and consensus statements have argued that older people should be encouraged to consume greater quantities of protein than the RDA (1.0–1.5 g/kg) [11,12,13,14]. Findings from observational studies are in line with these inferences, since higher protein consumption is associated with lower risk of frailty, loss of lean body mass, slow walking speed, dynapenia, and poor balance [15,16,17,18]. Nevertheless, there is a lack of direct evidence testing the proposed cut-off points for protein consumption. The few available studies have reported incongruent results regarding the association of protein intake and physical function [17,19,20,21]. However, to the best of our knowledge, meta-analyses have not been performed to determine the pool of results.

Therefore, the present work aimed at conducting a systematic review and meta-analysis of observational studies to investigate the association of relative protein intake and physical function in older adults.

## 2. Materials and Methods

We conducted a systematic review and meta-analysis of observational studies to assess the association between relative protein intake and physical function in older adults. The study was fully performed by investigators and no librarians were part of the team. This study complies with the criteria proposed by the Primary Reporting Items for Systematic Reviews and Meta-analyses (PRISMA) Statement [22], and the Meta-analysis of Observational Studies in Epidemiology (MOOSE) guidelines [23]. All data are available in the Open Science Framework at https://doi.org/10.17605/OSF.IO/JP5SB.

### 2.1. Eligibility Criteria

The inclusion criteria consisted of: (a) Observational studies, including cross-sectional and case-control studies, which investigated as primary or secondary outcome the association of relative protein intake and physical function in older adults. Longitudinal cohort studies were also included if crude baseline data were available; (b) participant age of 60 years or older; (c) direct assessment of at least one physical function domain (studies provided self-reported physical function were excluded); (d) provided the comparison of at least two groups with different relative protein intakes; (e) mean values and a measure of dispersion (standard deviation or confidence interval) were provided; (f) published studies (English language). We excluded randomized-clinical trials (RCTs), quasi-experimental, cross-over studies and any kind of investigation that examined the effects of a nutritional intervention associated or not with other interventions (e.g., physical exercise) on physical function. Studies that enrolled institutionalized participants or non-institutionalized participants with cognitive impairment and/or disorder, gastrointestinal and/or renal diseases, anorexia, cancer or any kind of condition that may directly impair protein metabolism (e.g., maple syrup urine disease, tyrosinemia) were also excluded. Sarcopenic and frailty older people were included. 

### 2.2. Search Strategy and Selection Criteria

Studies published on or before August 2018 were retrieved from the following three electronic databases by one investigator (H.J.C.J): (1) MEDLINE (PubMed interface); (2) the Cochrane Library (Wiley interface); (3) SCOPUS (Elsevier interface); (4) CINAHL (EBSCO interface); (5) AgeLine (EBSCO interface); and (6) EMBASE (EBSCO interface). Reference lists for reviews and retrieved articles for additional studies were checked and citation searches on key articles were performed in Google Scholar and ResearchGate for additional reports. Initially, a search strategy was designed using keywords, MeSH terms, and free text words, such as *protein consumption*, *physical function*, *older adults*. Additionally, keywords and subject headings were exhaustively combined using Boolean operators. The complete search strategy used for the PubMed is shown in List S1. Only eligible full texts in English language were considered for review. Authors were contacted if necessary.

### 2.3. Data Extraction and Quality Assessment 

Titles and abstracts of retrieved articles were screened for eligibility by two researchers (H.J.C.-J. and B.R.). If an abstract did not provide enough information for evaluation, the full-text was retrieved. Disagreements were solved by a third reviewer (M.U.). Reviewers were not blinded to authors, institutions, or manuscript journals. Studies that provided data for more than two groups—for example, low, middle, high, and very high relative protein intake were also added—since the volunteers were not shared among the groups. Data extraction were independently performed by two reviewers (H.J.C.-J. and L.M.-T) using a standardized coding form. Disagreements were solved by a third reviewer (M.U.). Coded variables included methodological quality and the characteristics of the studies, including: Year, authors, country, study design, setting, sample size (*n*), age, prevalence of female, body mass index (BMI), lean mass, appendicular muscle mass, dietary intake assessment method, total protein intake, relative protein intake.

Afterwards, studies were allocated into four different groups (*low* (<0.8 g/kg/day), *middle* (0.8–0.99 g/kg/day), *high* (≥1.0 g/kg/day), and *very high* (≥1.2 g/kg/day) protein intake). These cutoffs were selected according to previous research. Indeed, longitudinal [24,25] and review [11,12,13,14] studies have arguing that older adults should consume at least 1.0 g/kg/day of protein (i.e., *high*) to maintain muscle mass and optimal physical functioning, so that values below the RDA (<0.8 g/kg/day) may be considered *low*, while values higher than the RDA, but lower than the recommended for these aforementioned studies may be considered *middle*. In addition, some evidence has proposed that a minimum of 1.2 g/kg/day of protein should be consumed by older adults in attempt to avoid poor health-related outcomes and maintain functional performance, regardless the presence of chronic diseases [26,27]. In this sense, investigations that showed a mean protein intake of at least 1.2 g/kg/day were allocated in the *very high* group.

The quality of reporting for each study was performed by two researchers (H.J.C.-J. and L.M.-T) using the Strengthening the Reporting of Observational Studies in Epidemiology (STROBE) instrument [28]. The agreement rate between reviewers was *κ* = 0.96 for quality assessment.

### 2.4. Statistical Analysis

Meta-analyses were conducted using Revman V.5. Effect size (ES) were measured using standard mean difference (SMD) and mean difference and are reported with 95% confidence intervals (95% CI). SMD was used in the comparisons between *High protein intake* and *Very high protein intake* versus *Low protein intake* in relation to *Mobility* and *Lower limb physical functioning*, respectively, since the investigations assessed the same outcome, but using different tools. However, the mean difference was used in the remaining comparisons, since all the other studies used the same outcome. If the required outcome metric was not reported in the study, values were calculated using available data. Due to the different characteristics of the included studies, a random-effect model was used to calculate the pooled ES. Heterogeneity across studies was tested using Q-statistics and I^2^ index was used to assess inconsistency [29]. The I^2^ index was classified as not important (0–40%), moderate (30–60%), substantial (50–90%), and considerable (75–100%).

## 3. Results

### 3.1. Characteristics and Quality of Included Studies

**Figure 1:**
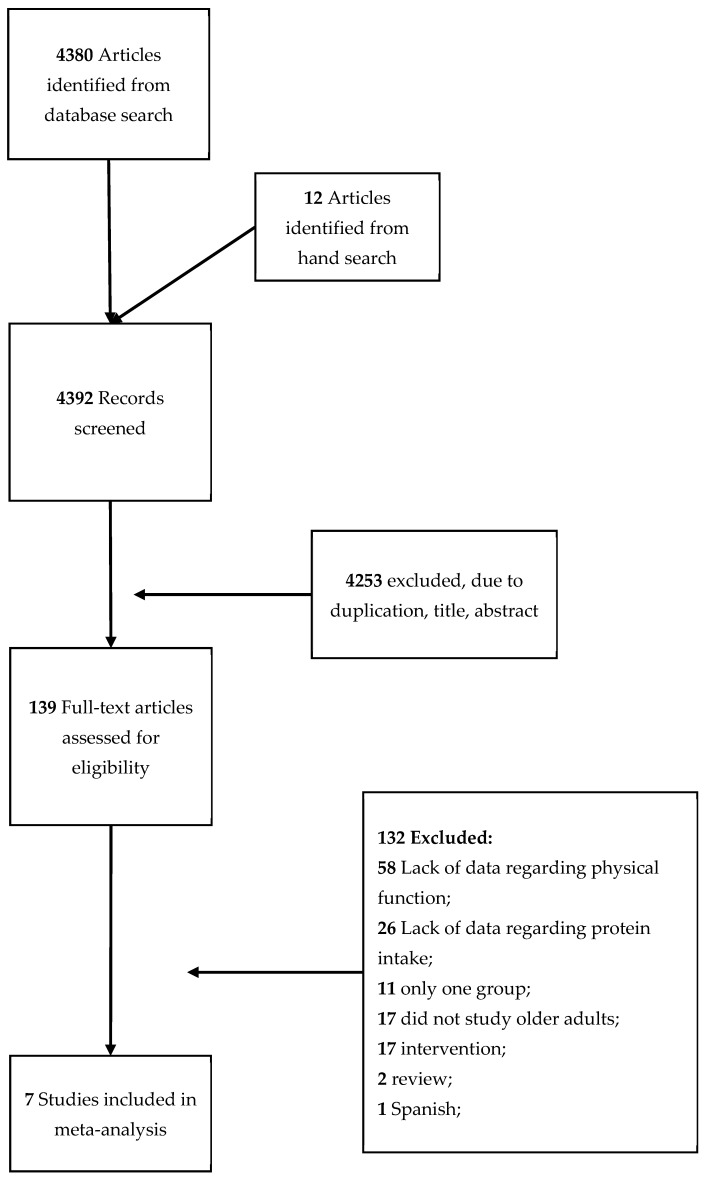
Flowchart of the present study.

Table 1 provides a general description of the included studies. Of the 4392 registers recovered from electronic databases and hand search, 4253 records were excluded based on duplicate data, title or abstract. One hundred thirty-nine studies were fully reviewed and assessed for eligibility. Finally, seven studies met the inclusion criteria (Figure 1).

Included studies were published between 2014 and 2018, the majority had a prospective longitudinal cohort design [17,30,31,32], while two had a cross-sectional design [20,33] and one study was a case-control [21]. Overall, a total of 8754 community-dwelling older adults from six different countries were included. Volunteers were characterized as healthy in three studies [17,31,34], post-menopausal in two studies [20,31], sarcopenic in one study [21], and diabetic in one study [32]. Mean age of the subjects ranged from 67.8 to 83.0 years, and the percentage of women among total subject population of various study groups varied from 10% to 100%. Mean BMI ranged from 23.7 kg/m^2^ to 29.5 kg/m^2^, so that one study investigated volunteers with normal BMI [34], while the other six studies investigated overweight individuals [17,20,21,31,32,33]. Limited information was available regarding the clinical characteristics of study participants. Nevertheless, osteoporosis, diabetes, hypertension, depression, rheumatoid arthritis, and heart diseases were diagnosed among the included individuals. Lean mass and appendicular skeletal muscle represented 55.8% and 24.4%, respectively, of the total weight. Twenty-nine percent of the volunteers reported an episode of fall in the 12 months before the investigations. Physical and functional evaluations included isometric handgrip strength (IHG), knee extensor strength, one-leg stance, usual walking speed (WS), chair rise, tandem walk speed, narrow walk speed, short physical performance battery (SPPB), and timed 8-foot walk. However, only IHG, WS, knee extensor strength, SPPB, and chair rise were included in the final analysis, due to availability of data. According to protein intake per kg of body weight, volunteers could be divided into four major groups: *Low* (<0.8 g/kg/day), *middle* (0.8–0.99 g/kg/day), *high* (≥1.0 g/kg/day), and *very high* (≥1.2 g/kg/day). Methods to evaluate dietary intake included 24-h dietary recall (28.5%), 3-day dietary intake record (28.5%), 4-day dietary intake record (14.3%), food frequency questionnaire (14.3%), and the Semi Quantitative-Food Frequency Questionnaire (SQFFQ) (14.3%).

Table 2 provides the general characteristics of the volunteers according to their relative protein intake. All groups presented similar mean age (~73 years). The lowest sample size was observed in the middle protein intake group, followed by the very high protein intake group, low protein intake group and high protein intake group. The groups presented a similar mean lean mass and mean appendicular mass. However, it is important to observe that High protein intake and Very high protein intake groups showed a higher percentage of lean mass when compared to Low protein intake and Middle protein intake groups. In addition, a greater performance in knee extensor strength and SPPB was observed in High protein intake and Very high protein intake groups when compared to Low protein intake group. Protein, carbohydrate and fat intake increased according to relative protein intake. It should be stressed that these parameters were not reported by all the investigations.

Study quality results are shown in Table S1, while the point by point analysis is shown in Table S2. The overall score ranged from 17 to 20. All studies reported the items required by the STROBE criteria in relation to the abstract (items 1 and 2), clarity of the outcomes (items 7 and 15), methods of assessment (item 8), handle of quantitative variables (item 11), statistical methods and analysis (items 12, 16), discussion (items 18–21), and funding (item 22). However, 14.2% of the studies failed to clearly state specific objectives, including any prespecified hypotheses (item 3), the main aim of the investigation (item 4), describe the setting, locations and relevant dates of recruitment and data collection (item 5) [25], give the characteristics of study participants (item 14); and report other analyses done—e.g., analyses of subgroups and interactions, and sensitivity analyses (item 17). In turn, 28.5% did not properly report the eligibility criteria, and the sources and methods of selection of participants (item 6), 71.4% did not describe any efforts to address potential sources of bias (item 9), 57.1% explained how the study size was arrived at (item 10) and reported numbers of individuals at each stage of study (item 13).

### 3.2. High Protein Intake verses Low Protein Intake

**Figure 2:**
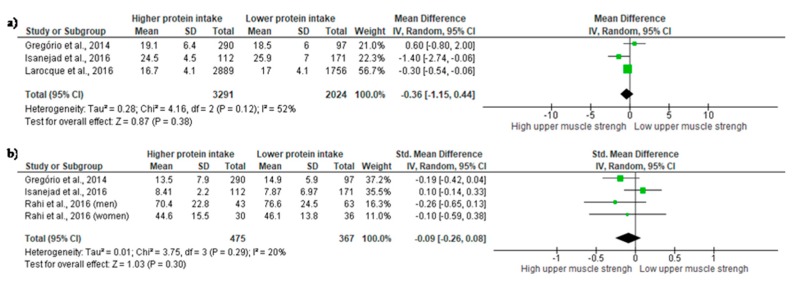
Mean difference in (**a**) *Upper-limb muscle strength* and Standardized mean difference in (**b**) *Lower-limb muscle strength* according to protein intake. Squares represent study-specific estimates; diamonds represent pooled estimates of random-effects meta-analyses.

A total of four studies provided information to investigate the association of high and low protein intake with physical function (Figure 2). It should be stressed, that Rahi et al. [32] provided their data according to gender, and the results are presented accordingly. *Upper-limb muscle strength*—Upper-limb muscle strength was measured by IHG in all studies. Three studies were added in the meta-analysis [17,20,31]. Results did not demonstrate significant differences in IHG between the groups, and a small non-significant ES was observed (ES = −0.36; 95% CI = −1.15 to 0.44, *p* = 0.38). Moderate heterogeneity was found across studies (χ^2^ = 4.16, df = 2, *p*= 0.12, I^2^= 52%) (Figure 2a). *Lower-limb muscle strength*—Lower-limb muscle strength was evaluated by chair-rise and knee extensor strength. A meta-analysis of three studies—but evaluating four subgroups—observed a small non-significant difference between groups (ES = −0.09; 95% CI = −0.26 to 0.08, *p* = 0.30). A not important heterogeneity was found across studies (χ^2^ = 3.75, df = 3, *p* = 0.29, I^2^ = 20%) (Figure 2b).

### 3.3. Mobility 

**Figure 3:**
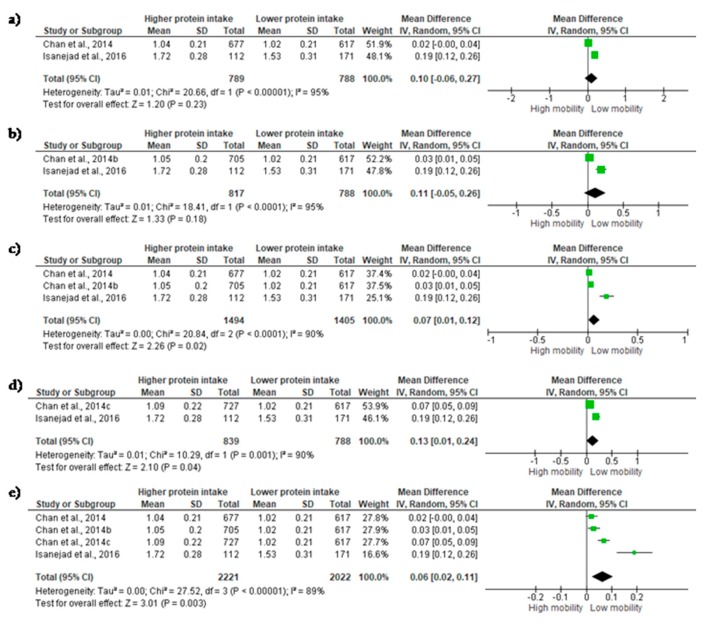
Mean differences in *Mobility* according to protein intake. (**a**) Chan et al., 2014, and Isanejad et al., 2016; (**b**) Chan et al., 2014b, and Isanejad et al., 2016; (**c**) Chan et al., 2014ab, and Isanejad et al., 2016; (**d**) Chan et al., 2014c, and Isanejad et al., 2016; (**e**) Chan et al., 2014abc, and Isanejad et al., 2016. Squares represent study-specific estimates; diamonds represent pooled estimates of random-effects meta-analyses.

Mobility was evaluated by 10-m WS [17] and 6-m WS [34]. In the study of Chan et al. [34], three out of four groups showed a high protein intake (≥1.0 g/kg/day). In this sense, groups will be mentioned as Chan et al., 2014, 2014b, and 2014c, according to relative protein intake. In addition, the groups were evaluated alone and grouped. A small ES were observed when the analysis was performed with Chan et al. [34] (1.0 g/kg/day) and Isanejad et al. [17] (ES = 0.10; 95% CI= −0.06 to 0.27, *p* = 0.23, χ^2^ = 20.66, df = 1, *p* < 0.00001, I^2^ = 95%) (Figure 3a), as well as with Chan et al. (2014b) (1.4 g/kg/day) and Isanejad et al. [15] (ES = 0.11; 95% CI = −0.05 to 0.26, *p* = 0.18, χ^2^ = 18.41, df = 1, *p* < 0.00001, I^2^ = 95%) (Figure 3b). The combination of the groups—Chan et al. (2014 and 2014b)—changed the results, so that a small and significant ES was observed (ES = 0.07; 95% CI = 0.01 to 0.12, *p* = 0.02, χ^2^ = 20.84, df = 2, *p* < 0.00001, I^2^ = 90%) (Figure 3c). Significant results were also observed when Chan et al. (2014c) was evaluated alone (ES = 0.13; 95% CI = 0.01 to 0.24, *p* = 0.04, χ^2^ = 10.29, df = 1, *p* = 0.01, I^2^ = 90%) (Figure 3d) and with the other groups (ES = 0.06; 95% CI = 0.02 to 0.11, *p* = 0.003, χ^2^ = 27.52, df = 3, *p* < 0.00001, I^2^ = 89%) (Figure 3e).

### 3.4. Middle Protein Intake verses High Protein Intake

**Figure 4:**
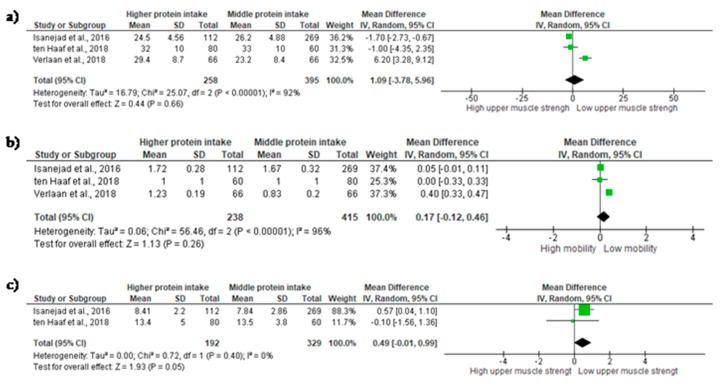
Mean difference in (**a**) *Upper-limb muscle strength*; (**b**) *Mobility*; and (**c**) *Lower-limb muscle strength* according to protein intake. Squares represent study-specific estimates; diamonds represent pooled estimates of random-effects meta-analyses.

A total of four studies provided information to investigate the association of high and middle protein intake with physical function (Figure 4). *Upper-limb muscle strength*—Upper-limb muscle strength was measured by IHG in all studies. Three studies were added in the meta-analysis [17,21,33]. Results did not demonstrate significant differences in IHG between groups, and a large non-significant ES was observed (ES = 1.09; 95% CI = −3.78 to 5.96, *p* = 0.66). A considerable heterogeneity was found across studies (χ^2^ = 25.07, df = 2, *p* = < 0.00001, I^2^ = 92%) (Figure 4a). *Mobility*—Mobility was evaluated in three studies. Pooling of results indicated a small and non-significant ES (ES = 0.17; 95% CI= −0.12 to 0.46, *p* = 0.26). A considerable heterogeneity was found across studies (χ^2^ = 56.46, df = 2, *p* = < 0.0001, I^2^ = 96%) (Figure 4b). *Lower-limb muscle strength*—Lower-limb muscle strength was evaluated by chair-rise in all studies. A meta-analysis of two studies observe a moderate non-significant difference between the groups (ES = 0.49; 95% CI= −0.01 to 0.99, *p* = 0.05). An insignificant heterogeneity was found across studies (χ^2^ = 0.72, df = 1, *p* = 0.40, I^2^ = 0%) (Figure 4c).

### 3.5. Very High Protein Intake verses Low Protein Intake

**Figure 5:**
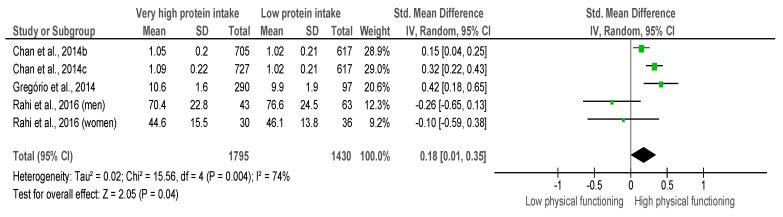
Standardized mean difference in *Lower-limb muscle functioning* according to protein intake. Squares represent study-specific estimates; diamonds represent pooled estimates of random-effects meta-analyses.

A total of five investigations provided information to investigate the association of *very high protein intake* and *low protein intake* with physical function (Figure 5). Due to the lack of available evidence, we did not divide the evaluation according to the type of physical assessment, as was performed above, and studies should assess at least one lower limb physical function to be included. The evaluations included knee extensor strength [32], SPPB [20], and walking speed [34]. Pooling of results indicated a small and significant ES (ES = 0.18; 95% CI = 0.01 to 0.35, *p* = 0.04). A considerable heterogeneity was found across studies (χ^2^ = 15.56, df = 4, *p* = 0.004, I^2^ = 74%).

## 4. Discussion

The present study was designed to investigate the available evidence regarding the association of relative protein intake and physical function in older adults. Findings of this investigation indicate that individuals with relatively very high (≥1.2 g/kg/day) and high (≥1.0 g/kg/day) protein intakes show higher mobility and lower limb physical functioning, respectively, in comparison to those with relative low protein (<0.80 g/kg/day) intake.

The assessment of study quality demonstrated that reports were of very good quality and scored between 17 and 20. The main bias associated with the studies was the lack of adequate description about the efforts to address potential sources of bias (item 9), the design of the study size (item 10), and the report regarding the number of participants in all the phases of the study (item 13).

Although in recent years several study groups have strongly recommended that older adults consume greater levels of protein intake than the RDA, there is a lack of direct evidence testing this hypothesis [11,13]. Several observational studies have demonstrated incongruent results, so that it is possible to observe null [19,33,34] and positive [17,20,21] associations between protein intake and physical function in older adults.

To the best of our knowledge, this is the first study that directly compared the physical function of older adults with different relative protein intakes. Our findings support at least partially the need to increase protein guidelines to above the current RDA in older adults, since the very high and high protein intake groups showed better muscular health when compared to the low protein intake group. The plausibility behind these findings is based on the anabolic resistance hypothesis, according to which the muscular anabolic response to appropriate stimulation would be blunted in advanced age (to review, see Calvani et al. [14]; Landi et al. [35]). This idea is supported by the observation that the aging muscle presents diminished muscle protein synthesis in response to small amount of essential amino acids (EAAs) [36], the key nutrient for the stimulation of protein synthesis. This would eventually lead to muscle catabolism, loss on lean body mass, dynapenia, and impairment on muscle function [35]. Higher availability of EAAs, mainly leucine, seems to be necessary to reverse overcome the anabolic resistance of muscle [37]. Therefore, the greater physical performance observed in the groups with higher protein intake levels (i.e., very high and high) might be ascribed to a larger EAAs availability. 

Although our findings demonstrated that very high and high protein intakes were associated with greater physical functioning in comparison to low protein intake, there were no differences between high and middle protein intake groups. These results are interesting and deserve concern because the *middle* group represented the level of protein intake recommended by the RDA.

The main motivation for considering changes from a minimum of 0.8 g/kg/day to 1.0 g/kg/day has been the findings of longitudinal studies that demonstrated preserved muscle mass [24] and lower risk of frailty [25] in older adults who had a protein intake ≥1.0 g/kg/day, as well as the evidence that showed a significant reduction on muscle mass of older adults who consumed the current RDA of protein for a long period [38]. However, no previous studies had directly comparing these proposed protein cutoffs, and the lack of significant differences between *high* and *middle* groups may occur, because the values of protein intake are similar, according to ten Haaf et al. [33]. 

Nonetheless, some researchers may argue that *very high* protein intake could be sufficient to elicit significant differences, since the studies of Vellas et al. [26] and Mustafa et al. [27] demonstrated that a very high protein intake was associated with a lower risk to poor health-related outcomes and physical disability. However, there was no available evidence to compare *very high* and *middle* protein intake groups. Taken together, these data suggest that a protein intake higher than 1.0 g/kg/day causes beneficial effects when compared to protein intake levels lower than 0.8 g/kg/day, but more studies are still necessary to precisely define the different effects of *very high* and *high* protein intakes in comparison to *middle* protein intake.

Conversely, from a practical point of view, the consumption of high protein intake by older adults has been the subject of intense scientific debate and a frequent concern of health professionals. Nowadays, has been accepted that older adults without a previous history of kidney disease show a lower risk of poor-health outcomes in response to high-protein diets [13,39]. However, although higher glomerular filtration rate seems to be a normal mechanism in response to the elevated amount of protein in the physiological system of patients with normal kidney function, an increased protein intake may collaborate to decline in the renal function of patients with a pre-existing renal disease [39]. Therefore, findings of the present study should be carefully extrapolated for other populations than healthy older adults.

On the other hand, data of the present study demonstrated that high protein intake was not associated with better performance on the IHG and chair rise when compared to low protein intake group. These findings support the inferences that a higher protein intake may be associated with better scores on some, but not all physical tests [19].

One possible explanation for these results is that a greater intake of protein might promote better functioning of systems other than the neuromuscular system. It should be stressed that the performance on the IHG and chair-rise seems to be mainly dictated by the neuromuscular system. On the other hand, walking ability needs a larger integration among the body systems in comparison with sit and stand up or tightening an object. Indeed, walking is a complex activity involving a variety of neural process (e.g., sensory, cortical cognitive, temporal) [40,41], cerebral and peripheral vascular beds [42,43], as well as lung [44], cardiac and muscular functions [45], to list a few. Consequently, walking ability represents the functioning of multiple organ systems instead of just one system [46], and marked disturbances in gait pattern may occur in response to cardiovascular, neurological and neuromuscular pathologies [40,41].

Regarding the relationship between protein intake and neural functioning, for example, evidence has demonstrated that an insufficient protein intake may impair spatial learning and memory and cause brain atrophy [47], while high protein intake decreases markers of oxidative stress (lipid peroxidation) in the brain of rats [48], and is associated with low levels of insoluble amyloid-β protein (Aβ) in older adults [49]. In addition, a systematic review showed that protein intake was positively associated with cognitive function in older adults [50]. Furthermore, increased protein intake may cause changes in the vessel wall structure and in cardiovascular control exerted by the central nervous system, consequently mediating the negative association between protein intake and blood pressure [51,52].

Physical activity levels [33], vitamin intake [31], inflammation [15], mood disorders [53], and the prevalence of chronic conditions (e.g., sarcopenia) [17] may also affect the relationship between protein intake and physical function. In the study by Isanejad et al. [17], for example, higher protein intake and physical function were significantly associated in non-sarcopenic, but not in sarcopenic older women. These inferences are in keeping with the hypothesis that individuals suffering from illness, physical stress, sarcopenia and/or frailty may require higher protein levels (1.2–1.5 g/kg) than healthy older adults [11,12,14]. In the present investigation, a considerable heterogeneity (I^2^) was observed in most of the studies. Although we tried to explore heterogeneity among the studies performing the analysis with random effects, the investigations did not offer sufficient details about the samples, as indicated in the quality assessment and food intake limiting the analysis of subgroups and meta-regression (see Table 2). Therefore, our results should be taken with caution and should be confirmed with further studies.

In this context, future studies aimed at investigating the association of protein intake and physical function should collect a number of data allowing better inferences and an inclusion in future systematic reviews and meta-analysis, including total and appendicular muscle mass, the prevalence of morbidities, frailty and sarcopenia assessment, physical activity levels, and an extensive report on food consumption (e.g., amino acid content, protein source) and not just the consumption of macronutrients. Other limitations of the present study include the lack of comparison between low and middle protein intake, as well as very high and middle protein intake (due to the lack of available data), and the use of the mean protein intake to identify the groups.

In relation to the latter, we allocated the groups mentioned in the studies into low, middle, high, and very high according to the mean protein intake reported. Nevertheless, it is possible that some individuals showed higher or lower protein intake levels. One possible way to solve this problem would be that future studies designed the groups based on proposed cut-offs for older adults [11,12,14], instead of separatrix measures (e.g., quartiles), since a low quartile does not necessarily represent a low protein intake.

## 5. Conclusions

In conclusion, findings of the present study indicate that a very high (≥1.2 g/kg/day) and high protein intake (≥1.0 g/kg/day) are associated with better lower-limb physical performance when compared to low protein (<0.80 g/kg/day) intake in community-dwelling older adults. These findings add evidence regarding the potential need to increase protein guidelines to above the current recommendations. However, large randomized clinical trials are needed to confirm the addictive effects of high-protein diets (≥1.0 g/kg/day) in comparison to the current recommendations on physical functioning.

## Figures and Tables

**Table 1:** General description of the included studies.

Year	Authors	Country	Study Design	Population	Setting	Sample Size	Age	Female (%)	BMI	Lean Mass	Appendicular Muscle Mass	Dietary Intake Assessment Method	Total Protein Intake (g/Day)	STROBE Score
2018	ten Haaf et al.	Netherland	Cross-sectional	Healthy	Community-dwelling	HP: 80; LP: 60	83.0	10	26.1	—	—	24-h dietary recall	HP: 89.5; LP: 64.7	19
2016	Isanejad et al.	Finland	Longitudinal	Healthy	Community-dwelling	HP = 112; MP = 269; LP = 171	67.8	100	26.6	HP: 41.3, 16.4, 6.5; MP:40.1, 15.9, 6.7; LP: 39.1, 15.6, 6.6	—	3-day dietary intake record	HP: 83.4; MP:65.0; LP: 51.4	20
2016	Rahi et al.	Canada	Longitudinal	Diabetic	Community-dwelling	HP: 73; LP: 99	75.0	62	29.5	—	—	24-h dietary recall	HP: 91; LP: 64.3	20
2015	Larocque et al.	United States	Longitudinal	Post-menopausal women	Community-dwelling	LP = 1756; HP = 2889	80.1	100	26.8	—	—	Food frequency questionnaire	LP = 42.6; HP = 71.6	17
2015	Verlaan et al.	United Kingdom	Case-control	Sarcopenic and non-sarcopenic	Community-dwelling	Sarcopenic: 66; Non-sarcopenic: 66	71.1	39	26.1	—	Sarcopenic:19.0; Non-sarcopenic: 20.4	3-day dietary intake record	Sarcopenic: 72.5; Non-sarcopenic: 75.3	19
2014	Chan et al.	China	Longitudinal	Healthy	Community-dwelling	LP = 617; MP =677; HP = 705; HP2 = 727	71.6	49.8	23.7	—	—	Semi Quantitative-Food Frequency Questionnaire (SQFFQ)	—	19
2014	Gregorio et al.	United States	Cross-sectional	Post-menopausal women	Community-dwelling	LP = 97; HP = 290	73.0	100	27.4	LP = 40.7; HP = 38.2	LP = 17.0; HP = 15.9	4-day dietary intake record	LP = 49.7; HP = 79.7	20

BMI = body mass index; HP = high protein; MP = middle protein; LP = low protein.

**Table 2:** Characteristics of the volunteers according to relative protein intake *.

	*Low Protein Intake (0.67)*	*Middle Protein Intake (0.88)*	*High Protein Intake (1.3)*	*Very High Protein Intake (1.5)*
Variables	*n* = 2641	*n* = 395	*n* = 5619	*n* = 1145
**Anthropometric characteristics**				
Age (years)	73.8	74.0	74.6	73.5
BMI (kg/m^2^)	29.1	26.7	27	27.1
Lean Mass (kg) (% in relation to weight)	41.0 (53)	40.1 (56.1)	38.7 (58.7)	38.2 (58.1)
Appendicular Muscle Mass (kg) (% in relation to weight)	—	19.0 (25.5)	20.4 (24.7)	15.9 (24.2)
**Physical functional tests**				
IHG (kg)	20.4	27.5	24.3	19.1
Knee Extensor Strength (lb)	54.5	44.5	52.1	57.5
One-Leg Stand (s)	13.5	19.3	18.4	15.3
Chair Rises (s)	11.4	10.6	11.8	13.5
Tandem Walk Speed for 6 m (m/s)	0.30	0.34	0.33	—
Usual Walking Speed (m/s)	1.1	1.2	1.2	1.07
SPPB (points)	9.9	9.0	11.0	10.6
Timed 8-Foot Walk (m/s)	1	—	1.1	1.1
**Dietary factors**				
Protein (g/day)	58.8	67.4	85.4	87.2
Carbohydrate (g/day)	162.6	199.8	215.9	220.6
Fat (g/day)	43.6	58.6	64.4	—

BMI = body mass index; IHG = Isometric handgrip; SPPB = Short physical performance battery (i.e., combination of results in gait speed, chair stand e balance tests; The final score ranged from 0 (worst performance) to 12 (best performance). * Information was not available by all the included investigations.

## Supplementary Materials

The following are available online at http://www.mdpi.com/2072-6643/10/9/1330/s1, List S1: The complete search strategy used for the PubMed. Table S1: Quality assessment analysis, Table S2: Individual quality assessment analysis of each included study.

## References

[1] Cruz-Jentoft A.J., Baeyens J.P., Bauer J.M., Boirie Y., Cederholm T., Landi F., Martin F.C., Michel J.-P., Rolland Y., Schneider S.M. (2010). Sarcopenia: European consensus on definition and diagnosis: Report of the European working group on sarcopenia in older people. Age Ageing.

[2] Diz J.B.M., Leopoldino A.A.O., Moreira B.D.S., Henschke N., Dias R.C., Pereira L.S.M., Oliveira V.C. (2017). Prevalence of sarcopenia in older Brazilians: A systematic review and meta-analysis. Geriatr. Gerontol. Int..

[3] Coelho Júnior H.J., Aguiar S.D.S., Gonçalves I.D.O., Sampaio R.A.C., Uchida M.C., Moraes M.R., Asano R.Y. (2015). Sarcopenia is associated with high pulse pressure in older women. J. Aging Res..

[4] Kim J.H., Lim S., Choi S.H., Kim K.M., Yoon J.W., Kim K.W., Lim J.-Y., Park K.S., Jang H.C., Kritchevsky S. (2014). Sarcopenia: An independent predictor of mortality in community-dwelling older Korean men. J. Gerontol. Ser. A.

[5] Brown J.C., Harhay M.O., Harhay M.N. (2016). Sarcopenia and mortality among a population-based sample of community-dwelling older adults. J. Cachexia. Sarcopenia Muscle.

[6] Benjumea A.-M., Curcio C.-L., Duque G., Gómez F. (2018). Dynapenia and sarcopenia as a risk factor for disability in a falls and fractures clinic in older persons. Open Access Maced. J. Med. Sci..

[7] Martone A.M., Marzetti E., Calvani R., Picca A., Tosato M., Santoro L., Di Giorgio A., Nesci A., Sisto A., Santoliquido A. (2017). Exercise and protein intake: A synergistic approach against sarcopenia. BioMed Res. Int..

[8] Marzetti E., Calvani R., Tosato M., Cesari M., Di Bari M., Cherubini A., Collamati A., D’Angelo E., Pahor M., Bernabei R. (2017). SPRINTT consortium sarcopenia: An overview. Aging Clin. Exp. Res..

[9] Nutrient Reference Values. https://www.nrv.gov.au/nutrients/protein.

[10] Protein—British Nutrition Foundation. https://www.nutrition.org.uk/nutritionscience/nutrients-food-and-ingredients/protein.html?start=1.

[11] Volpi E., Campbell W.W., Dwyer J.T., Johnson M.A., Jensen G.L., Morley J.E., Wolfe R.R. (2013). Is the optimal level of protein intake for older adults greater than the recommended dietary allowance?. J. Gerontol. Ser. A Biol. Sci. Med. Sci..

[12] Landi F., Calvani R., Tosato M., Martone A.M., Ortolani E., Savera G., D’Angelo E., Sisto A., Marzetti E. (2016). Protein intake and muscle health in old age: From biological plausibility to clinical evidence. Nutrients.

[13] Bauer J., Biolo G., Cederholm T., Cesari M., Cruz-Jentoft A.J., Morley J.E., Phillips S., Sieber C., Stehle P., Teta D. (2013). Evidence-based recommendations for optimal dietary protein intake in older people: A position paper from the prot-age study group. J. Am. Med. Dir. Assoc..

[14] Calvani R., Miccheli A., Landi F., Bossola M., Cesari M., Leeuwenburgh C., Sieber C.C., Bernabei R., Marzetti E. (2013). Current nutritional recommendations and novel dietary strategies to manage sarcopenia. J. Frailty Aging.

[15] Bartali B., Frongillo E.A., Bandinelli S., Lauretani F., Semba R.D., Fried L.P., Ferrucci L. (2006). Low nutrient intake is an essential component of frailty in older persons. J. Gerontol. A. Biol. Sci. Med. Sci..

[16] Lana A., Rodriguez-Artalejo F., Lopez-Garcia E. (2015). Dairy consumption and risk of frailty in older adults: A prospective cohort study. J. Am. Geriatr. Soc..

[17] Isanejad M., Mursu J., Sirola J., Kröger H., Rikkonen T., Tuppurainen M., Erkkilä A.T. (2016). Dietary protein intake is associated with better physical function and muscle strength among elderly women. Br. J. Nutr..

[18] Houston D.K., Schwartz A.V., Cauley J.A., Tylavsky F.A., Simonsick E.M., Harris T.B., De Rekeneire N., Schwartz G.G., Kritchevsky S.B. (2008). Serum parathyroid hormone levels predict falls in older adults with diabetes mellitus. J. Am. Geriatr. Soc..

[19] Farsijani S., Payette H., Morais J.A., Shatenstein B., Gaudreau P., Chevalier S. (2017). Even mealtime distribution of protein intake is associated with greater muscle strength, but not with 3-y physical function decline, in free-living older adults: The quebec longitudinal study on nutrition as a determinant of successful aging (NuAge study). Am. J. Clin. Nutr..

[20] Gregorio L., Brindisi J., Kleppinger A., Sullivan R., Mangano K.M., Bihuniak J.D., Kenny A.M., Kerstetter J.E., Insogna K.L. (2014). Adequate dietary protein is associated with better physical performance among post-menopausal women 60–90 years. J. Nutr. Health Aging.

[21] Verlaan S., Aspray T.J., Bauer J.M., Cederholm T., Hemsworth J., Hill T.R., McPhee J.S., Piasecki M., Seal C., Sieber C.C. (2017). Nutritional status, body composition, and quality of life in community-dwelling sarcopenic and non-sarcopenic older adults: A case-control study. Clin. Nutr..

[22] Liberati A., Altman D.G., Tetzlaff J., Mulrow C., Gøtzsche P.C., Ioannidis J.P.A., Clarke M., Devereaux P.J., Kleijnen J., Moher D. (2009). The PRISMA statement for reporting systematic reviews and meta-analyses of studies that evaluate health care interventions: Explanation and elaboration. PLoS Med..

[23] Stroup D.F., Berlin J.A., Morton S.C., Olkin I., Williamson G.D., Rennie D., Moher D., Becker B.J., Sipe T.A., Thacker S.B. (2000). Meta-analysis of observational studies in epidemiology: A proposal for reporting. JAMA.

[24] Houston D.K., Nicklas B.J., Ding J., Harris T.B., Tylavsky F.A., Newman A.B., Lee J.S., Sahyoun N.R., Visser M., Kritchevsky S.B. (2008). Health ABC study dietary protein intake is associated with lean mass change in older, community-dwelling adults: The health, aging, and body composition (Health ABC) study. Am. J. Clin. Nutr..

[25] Beasley J.M., Lacroix A.Z., Neuhouser M.L., Huang Y., Tinker L., Woods N., Michael Y., Curb J.D., Prentice R.L. (2010). Protein intake and incident frailty in the women’s health initiative observational study. J. Am. Geriatr. Soc..

[26] Vellas B.J., Hunt W.C., Romero L.J., Koehler K.M., Baumgartner R.N., Garry P.J. (1997). Changes in nutritional status and patterns of morbidity among free-living elderly persons: A 10-year longitudinal study. Nutrition.

[27] Mustafa J., Ellison R.C., Singer M.R., Bradlee M.L., Kalesan B., Holick M.F., Moore L.L. (2018). Dietary protein and preservation of physical functioning among middle-aged and older adults in the framingham offspring study. Am. J. Epidemiol..

[28] Von Elm E., Altman D.G., Egger M., Pocock S.J., Gøtzsche P.C., Vandenbroucke J.P., STROBE Initiative (2007). The strengthening the reporting of observational studies in epidemiology (STROBE) statement: Guidelines for reporting observational studies. Lancet.

[29] Green S., Higgins J. (2005). Cochrane Handbook for Systematic Reviews of Interventions.

[30] Chan R., Leung J., Woo J. (2015). Dietary patterns and risk of frailty in Chinese community-dwelling older people in Hong Kong: A prospective cohort study. Nutrients.

[31] Larocque S.C., Kerstetter J.E., Cauley J.A., Insogna K.L., Ensrud K., Lui L.-Y., Allore H.G. (2015). Dietary protein and vitamin D intake and risk of falls: A secondary analysis of postmenopausal women from the study of osteoporotic fractures. J. Nutr. Gerontol. Geriatr..

[32] Rahi B., Morais J.A., Gaudreau P., Payette H., Shatenstein B. (2016). Energy and protein intakes and their association with a decline in functional capacity among diabetic older adults from the NuAge cohort. Eur. J. Nutr..

[33] Ten Haaf D., van Dongen E., Nuijten M., Eijsvogels T., de Groot L., Hopman M. (2018). Protein intake and distribution in relation to physical functioning and quality of life in community-dwelling elderly people: acknowledging the role of physical activity. Nutrients.

[34] Chan R., Leung J., Woo J., Kwok T. (2014). Associations of dietary protein intake on subsequent decline in muscle mass and physical functions over four years in ambulant older Chinese people. J. Nutr. Health Aging.

[35] Landi F., Calvani R., Cesari M., Tosato M., Martone A.M., Ortolani E., Savera G., Salini S., Sisto A., Picca A. (2018). Sarcopenia: An overview on current definitions, diagnosis and treatment. Curr. Protein Pept. Sci..

[36] Katsanos C.S., Kobayashi H., Sheffield-Moore M., Aarsland A., Wolfe R.R. (2005). Aging is associated with diminished accretion of muscle proteins after the ingestion of a small bolus of essential amino acids. Am. J. Clin. Nutr..

[37] Katsanos C.S., Kobayashi H., Sheffield-Moore M., Aarsland A., Wolfe R.R. (2006). A high proportion of leucine is required for optimal stimulation of the rate of muscle protein synthesis by essential amino acids in the elderly. Am. J. Physiol. Metab..

[38] Campbell W.W., Trappe T.A., Wolfe R.R., Evans W.J. (2001). The recommended dietary allowance for protein may not be adequate for older people to maintain skeletal muscle. J. Gerontol. A. Biol. Sci. Med. Sci..

[39] Martin W.F., Armstrong L.E., Rodriguez N.R. (2005). Dietary protein intake and renal function. Nutr. Metab..

[40] Hamacher D., Herold F., Wiegel P., Hamacher D., Schega L. (2015). Brain activity during walking: A systematic review. Neurosci. Biobehav. Rev..

[41] Paraskevoudi N., Balcı F., Vatakis A. (2018). “Walking” through the sensory, cognitive, and temporal degradations of healthy aging. Ann. N. Y. Acad. Sci..

[42] EI Khoudary S.R., Chen H.-Y., Barinas-Mitchell E., McClure C., Selzer F., Karvonen-Gutierrez C., Jackson E.A., Ylitalo K.R., Sternfeld B. (2015). Simple physical performance measures and vascular health in late midlife women: The study of women’s health across the nation. Int. J. Cardiol..

[43] Su N., Zhai F.-F., Zhou L.-X., Ni J., Yao M., Li M.-L., Jin Z.-Y., Gong G.-L., Zhang S.-Y., Cui L.-Y. (2017). Cerebral small vessel disease burden is associated with motor performance of lower and upper extremities in community-dwelling populations. Front. Aging Neurosci..

[44] Nolan C.M., Maddocks M., Maher T.M., Canavan J.L., Jones S.E., Barker R.E., Patel S., Jacob J., Cullinan P., Man W.D.-C. (2018). Phenotypic characteristics associated with slow gait speed in idiopathic pulmonary fibrosis. Respirology.

[45] Vandervoort A.A. (2002). Aging of the human neuromuscular system. Muscle Nerve.

[46] Rosso A.L., Sanders J.L., Arnold A.M., Boudreau R.M., Hirsch C.H., Carlson M.C., Rosano C., Kritchevsky S.B., Newman A.B. (2015). Multisystem physiologic impairments and changes in gait speed of older adults. J. Gerontol. Ser. A Biol. Sci. Med. Sci..

[47] Reyes-Castro L.A., Padilla-Gómez E., Parga-Martínez N.J., Castro-Rodríguez D.C., Quirarte G.L., Díaz-Cintra S., Nathanielsz P.W., Zambrano E. (2018). Hippocampal mechanisms in impaired spatial learning and memory in male offspring of rats fed a low-protein isocaloric diet in pregnancy and/or lactation. Hippocampus.

[48] Madani Z., Malaisse W.J., Ait-Yahia D. (2015). A comparison between the impact of two types of dietary protein on brain glucose concentrations and oxidative stress in high fructose-induced metabolic syndrome rats. Biomed. Rep..

[49] Fernando W.M.A.D., Rainey-Smith S.R., Gardener S.L., Villemagne V.L., Burnham S.C., Macaulay S.L., Brown B.M., Gupta V.B., Sohrabi H.R., Weinborn M. (2018). Associations of dietary protein and fiber intake with brain and blood amyloid-β. J. Alzheimer’s Dis..

[50] Koh F., Charlton K., Walton K., McMahon A.-T. (2015). Role of dietary protein and thiamine intakes on cognitive function in healthy older people: A systematic review. Nutrients.

[51] Liu R., Dang S., Yan H., Wang D., Zhao Y., Li Q., Liu X. (2013). Association between dietary protein intake and the risk of hypertension: A cross-sectional study from rural western China. Hypertens. Res..

[52] Tielemans S.M.A.J., Altorf-van der Kuil W., Engberink M.F., Brink E.J., van Baak M.A., Bakker S.J.L., Geleijnse J.M. (2013). Intake of total protein, plant protein and animal protein in relation to blood pressure: A meta-analysis of observational and intervention studies. J. Hum. Hypertens..

[53] Guligowska A., Pigłowska M., Fife E., Kostka J., Sołtysik B.K., Kroc Ł., Kostka T. (2016). Inappropriate nutrients intake is associated with lower functional status and inferior quality of life in older adults with depression. Clin. Interv. Aging.

