# Supplementary material for: Relative Protein Intake and Physical Function in Older Adults: A Systematic Review and Meta-Analysis of Observational Studies
Source: Nutrients. 2018 Sep 19;10(9):1330. doi: 10.3390/nu10091330 (PMC6163569; doi:10.3390/nu10091330)
Supplement: Supplementary file 1 [file nutrients-10-01330-s001.zip › nutrients-339204-supplementary-final/List S1-proofreading.docx]

**List S1.** The complete search strategy used for the PubMed

1. Protein consumption AND physical function AND older adults

(("proteins"[MeSH Terms] OR "proteins"[All Fields] OR "protein"[All Fields]) AND ("economics"[MeSH Terms] OR "economics"[All Fields] OR "consumption"[All Fields])) AND (("physical examination"[MeSH Terms] OR ("physical"[All Fields] AND "examination"[All Fields]) OR "physical examination"[All Fields] OR "physical"[All Fields]) AND ("physiology"[Subheading] OR "physiology"[All Fields] OR "function"[All Fields] OR "physiology"[MeSH Terms] OR "function"[All Fields])) AND (older[All Fields] AND ("adult"[MeSH Terms] OR "adult"[All Fields] OR "adults"[All Fields]))

1. Protein consumption OR protein intake AND physical function AND older adults

(("proteins"[MeSH Terms] OR "proteins"[All Fields] OR "protein"[All Fields]) AND ("economics"[MeSH Terms] OR "economics"[All Fields] OR "consumption"[All Fields])) OR (("proteins"[MeSH Terms] OR "proteins"[All Fields] OR "protein"[All Fields]) AND intake[All Fields]) AND (("physical examination"[MeSH Terms] OR ("physical"[All Fields] AND "examination"[All Fields]) OR "physical examination"[All Fields] OR "physical"[All Fields]) AND ("physiology"[Subheading] OR "physiology"[All Fields] OR "function"[All Fields] OR "physiology"[MeSH Terms] OR "function"[All Fields])) AND (older[All Fields] AND ("adult"[MeSH Terms] OR "adult"[All Fields] OR "adults"[All Fields]))

1. Protein consumption AND muscle strength AND older adults

(("proteins"[MeSH Terms] OR "proteins"[All Fields] OR "protein"[All Fields]) AND ("economics"[MeSH Terms] OR "economics"[All Fields] OR "consumption"[All Fields])) AND ("muscle strength"[MeSH Terms] OR ("muscle"[All Fields] AND "strength"[All Fields]) OR "muscle strength"[All Fields]) AND (older[All Fields] AND ("adult"[MeSH Terms] OR "adult"[All Fields] OR "adults"[All Fields]))

1. Protein consumption AND muscle power AND older adults

(("proteins"[MeSH Terms] OR "proteins"[All Fields] OR "protein"[All Fields]) AND ("economics"[MeSH Terms] OR "economics"[All Fields] OR "consumption"[All Fields])) AND (("muscles"[MeSH Terms] OR "muscles"[All Fields] OR "muscle"[All Fields]) AND ("power (psychology)"[MeSH Terms] OR ("power"[All Fields] AND "(psychology)"[All Fields]) OR "power (psychology)"[All Fields] OR "power"[All Fields])) AND (older[All Fields] AND ("adult"[MeSH Terms] OR "adult"[All Fields] OR "adults"[All Fields]))

1. Protein consumption AND functional capacity AND older adults

(("proteins"[MeSH Terms] OR "proteins"[All Fields] OR "protein"[All Fields]) AND ("economics"[MeSH Terms] OR "economics"[All Fields] OR "consumption"[All Fields])) AND (functional[All Fields] AND capacity[All Fields]) AND (older[All Fields] AND ("adult"[MeSH Terms] OR "adult"[All Fields] OR "adults"[All Fields]))

1. Protein consumption AND aerobic capacity AND older adults

(("proteins"[MeSH Terms] OR "proteins"[All Fields] OR "protein"[All Fields]) AND ("economics"[MeSH Terms] OR "economics"[All Fields] OR "consumption"[All Fields])) AND (aerobic[All Fields] AND capacity[All Fields]) AND (older[All Fields] AND ("adult"[MeSH Terms] OR "adult"[All Fields] OR "adults"[All Fields]))

1. Protein consumption AND balance AND older adults

(("proteins"[MeSH Terms] OR "proteins"[All Fields] OR "protein"[All Fields]) AND ("economics"[MeSH Terms] OR "economics"[All Fields] OR "consumption"[All Fields])) AND ("Balance"[Journal] OR "balance"[All Fields]) AND (older[All Fields] AND ("adult"[MeSH Terms] OR "adult"[All Fields] OR "adults"[All Fields]))

1. Protein consumption AND walking speed AND older adults

(("proteins"[MeSH Terms] OR "proteins"[All Fields] OR "protein"[All Fields]) AND ("economics"[MeSH Terms] OR "economics"[All Fields] OR "consumption"[All Fields])) AND ("walking speed"[MeSH Terms] OR ("walking"[All Fields] AND "speed"[All Fields]) OR "walking speed"[All Fields]) AND (older[All Fields] AND ("adult"[MeSH Terms] OR "adult"[All Fields] OR "adults"[All Fields]))

1. Protein consumption AND functional status AND older adults

(("proteins"[MeSH Terms] OR "proteins"[All Fields] OR "protein"[All Fields]) AND ("economics"[MeSH Terms] OR "economics"[All Fields] OR "consumption"[All Fields])) AND (functional[All Fields] AND status[All Fields]) AND (older[All Fields] AND ("adult"[MeSH Terms] OR "adult"[All Fields] OR "adults"[All Fields]))

1. Protein consumption AND muscle function AND older adults

(("proteins"[MeSH Terms] OR "proteins"[All Fields] OR "protein"[All Fields]) AND ("economics"[MeSH Terms] OR "economics"[All Fields] OR "consumption"[All Fields])) AND (("muscles"[MeSH Terms] OR "muscles"[All Fields] OR "muscle"[All Fields]) AND ("physiology"[Subheading] OR "physiology"[All Fields] OR "function"[All Fields] OR "physiology"[MeSH Terms] OR "function"[All Fields])) AND (older[All Fields] AND ("adult"[MeSH Terms] OR "adult"[All Fields] OR "adults"[All Fields]))

1. Protein consumption AND physical performance AND older adults

(("proteins"[MeSH Terms] OR "proteins"[All Fields] OR "protein"[All Fields]) AND ("economics"[MeSH Terms] OR "economics"[All Fields] OR "consumption"[All Fields])) AND (("physical examination"[MeSH Terms] OR ("physical"[All Fields] AND "examination"[All Fields]) OR "physical examination"[All Fields] OR "physical"[All Fields]) AND performance[All Fields]) AND (older[All Fields] AND ("adult"[MeSH Terms] OR "adult"[All Fields] OR "adults"[All Fields]))

1. Protein consumption AND sarcopenia AND older adults

(("proteins"[MeSH Terms] OR "proteins"[All Fields] OR "protein"[All Fields]) AND ("economics"[MeSH Terms] OR "economics"[All Fields] OR "consumption"[All Fields])) AND ("sarcopenia"[MeSH Terms] OR "sarcopenia"[All Fields]) AND (older[All Fields] AND ("adult"[MeSH Terms] OR "adult"[All Fields] OR "adults"[All Fields]))

1. Protein consumption AND frailty AND older adults

(("proteins"[MeSH Terms] OR "proteins"[All Fields] OR "protein"[All Fields]) AND ("economics"[MeSH Terms] OR "economics"[All Fields] OR "consumption"[All Fields])) AND ("frailty"[MeSH Terms] OR "frailty"[All Fields]) AND (older[All Fields] AND ("adult"[MeSH Terms] OR "adult"[All Fields] OR "adults"[All Fields]))

1. Dietary protein [TIAB] OR dietary pattern [TIAB] OR dietary factors [TIAB] AND physical function AND older adults

dietary protein[TIAB] OR dietary pattern[TIAB] OR dietary factors[TIAB] AND (("physical examination"[MeSH Terms] OR ("physical"[All Fields] AND "examination"[All Fields]) OR "physical examination"[All Fields] OR "physical"[All Fields]) AND ("physiology"[Subheading] OR "physiology"[All Fields] OR "function"[All Fields] OR "physiology"[MeSH Terms] OR "function"[All Fields])) AND (older[All Fields] AND ("adult"[MeSH Terms] OR "adult"[All Fields] OR "adults"[All Fields]))

1. Dietary protein[TIAB] OR dietary pattern[TIAB] OR dietary factors[TIAB] AND muscle strength AND older adults

dietary protein[TIAB] OR dietary pattern[TIAB] OR dietary factors[TIAB] AND ("muscle strength"[MeSH Terms] OR ("muscle"[All Fields] AND "strength"[All Fields]) OR "muscle strength"[All Fields]) AND (older[All Fields] AND ("adult"[MeSH Terms] OR "adult"[All Fields] OR "adults"[All Fields]))

1. Protein intake AND physical function AND older adults

(("proteins"[MeSH Terms] OR "proteins"[All Fields] OR "protein"[All Fields]) AND intake[All Fields]) AND (("physical examination"[MeSH Terms] OR ("physical"[All Fields] AND "examination"[All Fields]) OR "physical examination"[All Fields] OR "physical"[All Fields]) AND ("physiology"[Subheading] OR "physiology"[All Fields] OR "function"[All Fields] OR "physiology"[MeSH Terms] OR "function"[All Fields])) AND (older[All Fields] AND ("adult"[MeSH Terms] OR "adult"[All Fields] OR "adults"[All Fields]))

1. Protein intake AND functional capacity AND older adults

(("proteins"[MeSH Terms] OR "proteins"[All Fields] OR "protein"[All Fields]) AND intake[All Fields]) AND (functional[All Fields] AND capacity[All Fields]) AND (older[All Fields] AND ("adult"[MeSH Terms] OR "adult"[All Fields] OR "adults"[All Fields]))
