# Supplementary material for: Relative Protein Intake and Physical Function in Older Adults: A Systematic Review and Meta-Analysis of Observational Studies
Source: Nutrients. 2018 Sep 19;10(9):1330. doi: 10.3390/nu10091330 (PMC6163569; doi:10.3390/nu10091330)
Supplement: Supplementary file 1 [file nutrients-10-01330-s001.zip › nutrients-339204-supplementary-final/Table S1-proofreading.docx]

| **Table S1.** Quality assessment analysis. | | | | | | | | | | | | | | | | | | | | | | | | | |
| --- | --- | --- | --- | --- | --- | --- | --- | --- | --- | --- | --- | --- | --- | --- | --- | --- | --- | --- | --- | --- | --- | --- | --- | --- | --- |
|  | **Item No** | | | | | | | | | | | | | | | | | | | | | | |  | |
| **Study** | **1** | **2** | **3** | **4** | **5** | **6** | **7** | **8** | **9** | **10** | **11** | **12** | **13** | **14** | **15** | **16** | **17** | **18** | **19** | **20** | **21** | **22** | **Score** | |  |
| ten Haaf et al., 2018 | X | X | X | X |  |  | X | X | X |  | X | X |  | X | X | X | X | X | X | X | X | X | 19 | |  |
| Isanejad et al., 2016 | X | X | X | X | X | X | X | X | X |  | X | X |  | X | X | X | X | X | X | X | X | X | 20 | |  |
| Rahi et al., 2016 | X | X | X | X | X | X | X | X |  | X | X | X | X | X | X | X |  | X | X | X | X | X | 20 | |  |
| Larocque et al., 2015 | X | X | X | X | X |  | X | X |  |  | X | X |  |  | X | X | X | X | X | X | X | X | 17 | |  |
| Verlaan et al., 2015 | X | X | X | X | X | X | X | X |  |  | X | X | X | X | X | X | X | X | X | X | X | X | 19 | |  |
| Chan et al., 2014 | X | X |  |  | X | X | X | X |  | X | X | X | X | X | X | X | X | X | X | X | X | X | 19 | |  |
| Gregorio et al., 2014 | X | X | X | X | X | X | X | X |  | X | X | X |  | X | X | X | X | X | X | X | X | X | 20 | |  |

1. (a) Indicate the study’s design with a commonly used term in the title or the abstract; (b) provide in the abstract an informative and balanced summary of what was done and what was found;
2. Explain the scientific background and rationale for the investigation being reported;
3. State specific objectives, including any prespecified hypotheses;
4. Present key elements of study design early in the paper;
5. Describe the setting, locations, and relevant dates, including periods of recruitment, exposure, follow-up, and data collection;
6. Eligibility criteria, and the sources and methods of selection of participants;
7. Clearly define all outcomes, exposures, predictors, potential confounders, and effect modifiers. Give diagnostic criteria, if applicable;
8. For each variable of interest, give sources of data and details of methods of assessment (measurement). Describe comparability of assessment methods if there is more than one group;
9. Describe any efforts to address potential sources of bias;
10. Explain how the study size was arrived at;
11. Explain how quantitative variables were handled in the analyses. If applicable, describe which groupings were chosen and why;
12. Describe all statistical methods, including those used to control for confounding;
13. Report numbers of individuals at each stage of study—e.g., numbers potentially eligible, examined for eligibility, confirmed eligible, included in the study, completing follow-up, and analyzed;
14. Give characteristics of study participants (e.g., demographic, clinical, social) and information on exposures and potential confounders;
15. Report numbers of outcome events or summary measures;
16. Give unadjusted estimates and, if applicable, confounder-adjusted estimates and their precision (e.g., 95% confidence interval). Make clear which confounders were adjusted for and why they were included;
17. Report other analyses done—e.g., analyses of subgroups and interactions, and sensitivity analyses;
18. Summarize key results with reference to study objectives;
19. Discuss limitations of the study, taking into account sources of potential bias or imprecision. Discuss both direction and magnitude of any potential bias;
20. Give a cautious overall interpretation of results considering objectives, limitations, multiplicity of analyses, results from similar studies, and other relevant evidence;
21. Discuss the generalizability (external validity) of the study results;
22. Give the source of funding and the role of the funders for the present study and, if applicable, for the original study on which the present article is based.
