# Supplementary material for: Relative Protein Intake and Physical Function in Older Adults: A Systematic Review and Meta-Analysis of Observational Studies
Source: Nutrients. 2018 Sep 19;10(9):1330. doi: 10.3390/nu10091330 (PMC6163569; doi:10.3390/nu10091330)
Supplement: Supplementary file 1 [file nutrients-10-01330-s001.zip › nutrients-339204-supplementary-final/Table S2-proofreading.docx]

| **Table S2.** Individual quality assessment analysis of each included study. | | | | | |
| --- | --- | --- | --- | --- | --- |
| STROBE Statement—checklist of items that should be included in reports of observational studies | | | | | |
|  |  |  |  |  |  |
| **Article:** Adequate dietary protein is associated with better physical performance among post-menopausal women 60−90 years. | **Year:** 2014 |  |  |  |  |
| **Authors:**  Gregorio et al., |  |  |  |  |  |
|  |  |  |  |  |  |
|  | **Item No** | **Recommendation** | **Reported** | **Page (paragraph [line])** | **Note** |
| **Title and abstract** | **1** | *(a)* Indicate the study’s design with a commonly used term in the title or the abstract | X | 1 |  |
|  |  | *(b)* Provide in the abstract an informative and balanced summary of what was done and what was found | X | 1 |  |
| **Introduction** |  |  |  |  |  |
| *Background/rationale* | **2** | Explain the scientific background and rationale for the investigation being reported | X | 2–3 |  |
| *Objectives* | **3** | State specific objectives, including any prespecified hypotheses | X | 2–3 |  |
| **Methods** |  |  |  |  |  |
| *Study design* | **4** | Present key elements of study design early in the paper | X | 3 (2) |  |
| *Setting* | **5** | Describe the setting, locations, and relevant dates, including periods of recruitment, exposure, follow-up, and data collection | X | 3 (2) |  |
| *Participants* | **6** | *(a)* Cohort study—give the eligibility criteria, and the sources and methods of selection of participants. Describe methods of follow-up |  |  |  |
|  |  | Case-control study—give the eligibility criteria, and the sources and methods of case ascertainment and control selection. Give the rationale for the choice of cases and controls |  |  |  |
|  |  | Cross-sectional study—give the eligibility criteria, and the sources and methods of selection of participants | X | 3 (2) |  |
|  |  | *(b)* Cohort study—for matched studies, give matching criteria and number of exposed and unexposed |  |  |  |
|  |  | Case-control study—for matched studies, give matching criteria and the number of controls per case |  |  |  |
| *Variables* | **7** | Clearly define all outcomes, exposures, predictors, potential confounders, and effect modifiers. Give diagnostic criteria, if applicable | X | 3–4 |  |
| *Data sources/ measurement* | **8*** | For each variable of interest, give sources of data and details of methods of assessment (measurement). Describe comparability of assessment methods if there is more than one group | X | 3–4 |  |
| *Bias* | **9** | Describe any efforts to address potential sources of bias | — | — | Information was not available |
| *Study size* | **10** | Explain how the study size was decided | X | 3 (2) | Information was not available |
| *Quantitative variables* | **11** | Explain how quantitative variables were handled in the analyses. If applicable, describe which groupings were chosen and why | X | 3–4 |  |
| *Statistical methods* | **12** | *(a)* Describe all statistical methods, including those used to control for confounding | X | 4 (2) |  |
|  |  | *(b)* Describe any methods used to examine subgroups and interactions | X | 4 (2) |  |
|  |  | *(c)* Explain how missing data were addressed | — | — | Information was not available |
|  |  | *(d)* Cohort study—if applicable, explain how loss to follow-up was addressed |  |  |  |
|  |  | Case-control study—if applicable, explain how matching of cases and controls was addressed |  |  |  |
|  |  | Cross-sectional study—if applicable, describe analytical methods taking account of sampling strategy |  |  |  |
|  |  | *(e)* Describe any sensitivity analyses | X | 4 (2) |  |
| **Results** |  |  |  |  |  |
| *Participants* | **13*** | *(a)* Report numbers of individuals at each stage of study—e.g., numbers potentially eligible, examined for eligibility, confirmed eligible, included in the study, completing follow-up, and analysed | — | — | Information was not available |
|  |  | *(b)* Give reasons for non-participation at each stage | — | — | Information was not available |
|  |  | *(c)* Consider use of a flow diagram | — | — | Information was not available |
| *Descriptive data* | **14*** | *(a)* Give characteristics of study participants (e.g., demographic, clinical, social) and information on exposures and potential confounders | X | Table 1 |  |
|  |  | *(b)* Indicate number of participants with missing data for each variable of interest | — | — | Information was not available |
|  |  | *(c)* Cohort study—summarise follow-up time (e.g., average and total amount) |  |  |  |
| *Outcome data* | **15*** | Cohort study—report numbers of outcome events or summary measures over time |  |  |  |
|  |  | Case-control study—report numbers in each exposure category, or summary measures of exposure |  |  |  |
|  |  | Cross-sectional study—report numbers of outcome events or summary measures | X | Results section and Tables 2−3 |  |
| *Main results* | **16** | *(a)* Give unadjusted estimates and, if applicable, confounder-adjusted estimates and their precision (e.g., 95% confidence interval). Make it clear which confounders were adjusted for and why they were included | X | Table 3 |  |
|  |  | *(b)* Report category boundaries when continuous variables were categorized |  |  |  |
|  |  | *(c)* If relevant, consider translating estimates of relative risk into absolute risk for a meaningful time period |  |  |  |
| *Other analyses* | **17** | Report other analyses done—e.g., analyses of subgroups and interactions, and sensitivity analyses | X | Table 3 |  |
| **Discussion** |  |  |  |  |  |
| *Key results* | **18** | Summarise key results with reference to study objectives | X | 5 (5 [1–8]) |  |
| *Limitations* | **19** | Discuss limitations of the study, taking into account sources of potential bias or imprecision. Discuss both direction and magnitude of any potential bias | X | 5 (5 [1–8]), 6–7 |  |
| *Interpretation* | **20** | Give a cautious overall interpretation of results considering objectives, limitations, multiplicity of analyses, results from similar studies, and other relevant evidence | X | 5 (5 [1–8]), 6–7 |  |
| *Generalisability* | **21** | Discuss the generalisability (external validity) of the study results | X | 5 (5 [1–8]), 6–7 |  |
| **Other information** |  |  |  |  |  |
| *Funding* | **22** | Give the source of funding and the role of the funders for the present study and, if applicable, for the original study on which the present article is based | X | Acknowledgments |  |
| **TOTAL SCORE:** 20 |  |  |  |  |  |

| **STROBE Statement—checklist of items that should be included in reports of observational studies** |  |  |  |  |  |
| --- | --- | --- | --- | --- | --- |
|  |  |  |  |  |  |
| **Article:** Associations of dietary protein intake on subsequent decline in muscle mass and physical functions over four years in ambulant older Chinese people. | **Year:** 2014 |  |  |  |  |
| **Authors:**  Chan et al., |  |  |  |  |  |
|  |  |  |  |  |  |
|  | **Item No** | **Recommendation** | **Reported** | **Page (paragraph [line])** | **Note** |
| **Title and abstract** | **1** | *(a)* Indicate the study’s design with a commonly used term in the title or the abstract | X | 171 |  |
|  |  | *(b)* Provide in the abstract an informative and balanced summary of what was done and what was found | X | 171 |  |
| **Introduction** |  |  |  |  |  |
| *Background/rationale* | **2** | Explain the scientific background and rationale for the investigation being reported | X | 171 |  |
| *Objectives* | **3** | State specific objectives, including any prespecified hypotheses | — | — | The hypothesis was not well-described |
| **Methods** |  |  |  |  |  |
| *Study design* | **4** | Present key elements of study design early in the paper | — | — | The key elements of the study (e.g., design, evaluations) were not early mentioned |
| *Setting* | **5** | Describe the setting, locations, and relevant dates, including periods of recruitment, exposure, follow-up, and data collection | X | 172 (1) |  |
| *Participants* | **6** | *(a)* Cohort study—give the eligibility criteria, and the sources and methods of selection of participants. Describe methods of follow-up | X | 172 (1) |  |
|  |  | Case-control study—give the eligibility criteria, and the sources and methods of case ascertainment and control selection. Give the rationale for the choice of cases and controls |  |  |  |
|  |  | Cross-sectional study—give the eligibility criteria, and the sources and methods of selection of participants |  |  |  |
|  |  | *(b)* Cohort study—for matched studies, give matching criteria and number of exposed and unexposed |  |  |  |
|  |  | Case-control study—for matched studies, give matching criteria and the number of controls per case |  |  |  |
| *Variables* | **7** | Clearly define all outcomes, exposures, predictors, potential confounders, and effect modifiers. Give diagnostic criteria, if applicable | X | 172–173 |  |
| *Data sources/ measurement* | **8*** | For each variable of interest, give sources of data and details of methods of assessment (measurement). Describe comparability of assessment methods if there is more than one group | X | 172–173 |  |
| *Bias* | **9** | Describe any efforts to address potential sources of bias | — | — | Information was not available |
| *Study size* | **10** | Explain how the study size was decided | X | 172 (1 [8–13]) | Information was not available |
| *Quantitative variables* | **11** | Explain how quantitative variables were handled in the analyses. If applicable, describe which groupings were chosen and why | X | 172–173 |  |
| *Statistical methods* | **12** | *(a)* Describe all statistical methods, including those used to control for confounding | X | 173 (1 [1–8], 2 [1–16]), 174 [1–11] |  |
|  |  | *(b)* Describe any methods used to examine subgroups and interactions | X | 173 (1 [1–8], 2 [1–16]), 174 [1–11] |  |
|  |  | *(c)* Explain how missing data were addressed | X | — | Volunteers were excluded |
|  |  | *(d)* Cohort study—if applicable, explain how loss to follow-up was addressed | X | — | Volunteers were excluded |
|  |  | Case-control study—if applicable, explain how matching of cases and controls was addressed |  |  |  |
|  |  | Cross-sectional study—if applicable, describe analytical methods taking account of sampling strategy |  |  |  |
|  |  | *(e)* Describe any sensitivity analyses | X | 173 (1 [1–8], 2 [1–16]), 174 [1–11] |  |
| **Results** |  |  |  |  |  |
| *Participants* | **13*** | *(a)* Report numbers of individuals at each stage of study—e.g., numbers potentially eligible, examined for eligibility, confirmed eligible, included in the study, completing follow-up, and analysed | X | 174 (1 [1–10]) |  |
|  |  | *(b)* Give reasons for non-participation at each stage | — | — | Information was not available |
|  |  | *(c)* Consider use of a flow diagram | — | — | Information was not available |
| *Descriptive data* | **14*** | *(a)* Give characteristics of study participants (e.g., demographic, clinical, social) and information on exposures and potential confounders | X | Table 1 |  |
|  |  | *(b)* Indicate number of participants with missing data for each variable of interest | X | — | Volunteers with missing data were excluded |
|  |  | *(c)* Cohort study—summarise follow-up time (e.g., average and total amount) | X |  |  |
| *Outcome data* | **15*** | Cohort study—report numbers of outcome events or summary measures over time | X | Tables 1–4 |  |
|  |  | Case-control study—report numbers in each exposure category, or summary measures of exposure |  |  |  |
|  |  | Cross-sectional study—report numbers of outcome events or summary measures |  |  |  |
| *Main results* | **16** | *(a)* Give unadjusted estimates and, if applicable, confounder-adjusted estimates and their precision (e.g., 95% confidence interval). Make clear which confounders were adjusted for and why they were included | X | Tables 2–4 |  |
|  |  | *(b)* Report category boundaries when continuous variables were categorized | X | Tables 1–4 |  |
|  |  | *(c)* If relevant, consider translating estimates of relative risk into absolute risk for a meaningful time period |  |  |  |
| *Other analyses* | **17** | Report other analyses done—e.g., analyses of subgroups and interactions, and sensitivity analyses | X | Tables 1–4 |  |
| **Discussion** |  |  |  |  |  |
| *Key results* | **18** | Summarise key results with reference to study objectives | X | 174 (4 [1–4] |  |
| *Limitations* | **19** | Discuss limitations of the study, taking into account sources of potential bias or imprecision. Discuss both direction and magnitude of any potential bias | X | 174–176 |  |
| *Interpretation* | **20** | Give a cautious overall interpretation of results considering objectives, limitations, multiplicity of analyses, results from similar studies, and other relevant evidence | X | 174–176 |  |
| *Generalisability* | **21** | Discuss the generalisability (external validity) of the study results | X | 174–176 |  |
| **Other information** |  |  |  |  |  |
| *Funding* | **22** | Give the source of funding and the role of the funders for the present study and, if applicable, for the original study on which the present article is based | X | Acknowledgments |  |
| **TOTAL SCORE:** 19 |  |  |  |  |  |

| **STROBE Statement—checklist of items that should be included in reports of observational studies** |  |  |  |  |  |
| --- | --- | --- | --- | --- | --- |
|  |  |  |  |  |  |
| **Article:** Nutritional status, body composition, and quality of life in community-dwelling sarcopenic and non-sarcopenic older adults: A case-control study | **Year:** 2015 |  |  |  |  |
| **Authors:**  Verlaan et al., |  |  |  |  |  |
|  |  |  |  |  |  |
|  | **Item No** | **Recommendation** | **Reported** | **Page (paragraph [line])** | **Note** |
| **Title and abstract** | **1** | *(a)* Indicate the study’s design with a commonly used term in the title or the abstract | X | 1 |  |
|  |  | *(b)* Provide in the abstract an informative and balanced summary of what was done and what was found | X | 1 |  |
| **Introduction** |  |  |  |  |  |
| *Background/rationale* | **2** | Explain the scientific background and rationale for the investigation being reported | X | 2 (1, 2, 3) |  |
| *Objectives* | **3** | State specific objectives, including any prespecified hypotheses | X | 2 (1, 2, 3) | There was not an especifc setence about the hypothesis of the present study. However, volunteers made it clear across the introduction section |
| **Methods** |  |  |  |  |  |
| *Study design* | **4** | Present key elements of study design early in the paper | X | 2 (4 [1–3], 5 [1–20], [21–24], 6 [1–10], 7 [1–7] |  |
| *Setting* | **5** | Describe the setting, locations, and relevant dates, including periods of recruitment, exposure, follow-up, and data collection | X | 2 (4 [1–3], 5 [1–20], [21–24], 6 [1–10] |  |
| *Participants* | **6** | *(a)* Cohort study—give the eligibility criteria, and the sources and methods of selection of participants. Describe methods of follow-up |  |  |  |
|  |  | Case-control study—give the eligibility criteria, and the sources and methods of case ascertainment and control selection. Give the rationale for the choice of cases and controls | X | 2 (4 [1–3], 5 [1–20], [21–24], 6 [1–10] |  |
|  |  | Cross-sectional study—give the eligibility criteria, and the sources and methods of selection of participants |  |  |  |
|  |  | *(b)* Cohort study—for matched studies, give matching criteria and number of exposed and unexposed |  |  |  |
|  |  | Case-control study—for matched studies, give matching criteria and the number of controls per case | X | 2 (6 [1–10]) |  |
| *Variables* | **7** | Clearly define all outcomes, exposures, predictors, potential confounders, and effect modifiers. Give diagnostic criteria, if applicable | X | 2 (4 [1–3], 5 [1–20], [21–24], 6 [1–10], 7 [1–7] |  |
| *Data sources/ measurement* | **8*** | For each variable of interest, give sources of data and details of methods of assessment (measurement). Describe comparability of assessment methods if there is more than one group | X | 2–3 |  |
| *Bias* | **9** | Describe any efforts to address potential sources of bias | — | — | Information was not available |
| *Study size* | **10** | Explain how the study size was decided | — | — | Information was not available |
| *Quantitative variables* | **11** | Explain how quantitative variables were handled in the analyses. If applicable, describe which groupings were chosen and why | X | 2–3 |  |
| *Statistical methods* | **12** | *(a)* Describe all statistical methods, including those used to control for confounding | X | 3 (5 [1–3], [1–12]) |  |
|  |  | *(b)* Describe any methods used to examine subgroups and interactions | X | 3 (5 [1–3], [1–12]) |  |
|  |  | *(c)* Explain how missing data were addressed | — | — | Information was not available |
|  |  | *(d)* Cohort study—if applicable, explain how loss to follow-up was addressed |  |  |  |
|  |  | Case-control study—if applicable, explain how matching of cases and controls was addressed | X | 3 (5 [1–3], [1–12]) |  |
|  |  | Cross-sectional study—if applicable, describe analytical methods taking account of sampling strategy |  |  |  |
|  |  | *(e)* Describe any sensitivity analyses | — | — | Information was not available |
| **Results** |  |  |  |  |  |
| *Participants* | **13*** | *(a)* Report numbers of individuals at each stage of study—e.g., numbers potentially eligible, examined for eligibility, confirmed eligible, included in the study, completing follow-up, and analysed | X | 3 (6, 7, 8) |  |
|  |  | *(b)* Give reasons for non-participation at each stage | X | Figure 1 |  |
|  |  | *(c)* Consider use of a flow diagram | X | Figure 1 |  |
| *Descriptive data* | **14*** | *(a)* Give characteristics of study participants (e.g., demographic, clinical, social) and information on exposures and potential confounders | X | 3 (6, 7, 8) |  |
|  |  | *(b)* Indicate number of participants with missing data for each variable of interest | — | — | Information was not available |
|  |  | *(c)* Cohort study—summarise follow-up time (e.g., average and total amount) | X | Figure 1 |  |
| *Outcome data* | **15*** | Cohort study—report numbers of outcome events or summary measures over time |  |  |  |
|  |  | Case-control study—report numbers in each exposure category, or summary measures of exposure |  |  |  |
|  |  | Cross-sectional study—report numbers of outcome events or summary measures | X | Tables 1–4 |  |
| *Main results* | **16** | *(a)* Give unadjusted estimates and, if applicable, confounder-adjusted estimates and their precision (e.g., 95% confidence interval). Make clear which confounders were adjusted for and why they were included |  |  |  |
|  |  | *(b)* Report category boundaries when continuous variables were categorized | X | Tables 1–4, Figure 2 |  |
|  |  | *(c)* If relevant, consider translating estimates of relative risk into absolute risk for a meaningful time period |  |  |  |
| *Other analyses* | **17** | Report other analyses done—e.g., analyses of subgroups and interactions, and sensitivity analyses | X | Figure 2 |  |
| **Discussion** |  |  |  |  |  |
| *Key results* | **18** | Summarise key results with reference to study objectives | X | 4 (1 [1–7], [1–5]) |  |
| *Limitations* | **19** | Discuss limitations of the study, taking into account sources of potential bias or imprecision. Discuss both direction and magnitude of any potential bias | X | 4–7 |  |
| *Interpretation* | **20** | Give a cautious overall interpretation of results considering objectives, limitations, multiplicity of analyses, results from similar studies, and other relevant evidence | X | 4–7 |  |
| *Generalisability* | **21** | Discuss the generalisability (external validity) of the study results | X | 4–7 | Researchers suggested several inferences about their data across the discussion section. |
| **Other information** |  |  |  |  |  |
| *Funding* | **22** | Give the source of funding and the role of the funders for the present study and, if applicable, for the original study on which the present article is based | X | Authors contribution |  |
| **TOTAL SCORE:** 20 |  |  |  |  |  |

| **STROBE Statement—checklist of items that should be included in reports of observational studies** |  |  |  |  |  |
| --- | --- | --- | --- | --- | --- |
|  |  |  |  |  |  |
| **Article:** Dietary protein and vitamin D intake and risk of falls: A secondary analysis of postmenopausal women from the study of Osteoporotic fractures | **Year:** 2016 |  |  |  |  |
| **Authors:**  Laroque et al., |  |  |  |  |  |
|  |  |  |  |  |  |
|  | **Item No** | **Recommendation** | **Reported** | **Page (paragraph [line])** | **Note** |
| **Title and abstract** | **1** | *(a)* Indicate the study’s design with a commonly used term in the title or the abstract | X | 1–2 |  |
|  |  | *(b)* Provide in the abstract an informative and balanced summary of what was done and what was found | X | 1–2 |  |
| **Introduction** |  |  |  |  |  |
| *Background/rationale* | **2** | Explain the scientific background and rationale for the investigation being reported | X | 2–3 |  |
| *Objectives* | **3** | State specific objectives, including any prespecified hypotheses | X | 3 (1 [1–6]) |  |
| **Methods** |  |  |  |  |  |
| *Study design* | **4** | Present key elements of study design early in the paper | X | 3 (2, 3, 4) |  |
| *Setting* | **5** | Describe the setting, locations, and relevant dates, including periods of recruitment, exposure, follow-up, and data collection | X | 3 (2 [1–10]) |  |
| *Participants* | **6** | *(a)* Cohort study—give the eligibility criteria, and the sources and methods of selection of participants. Describe methods of follow-up | — | — | The eligibility criteria and methods of selection were not described |
|  |  | Case-control study—give the eligibility criteria, and the sources and methods of case ascertainment and control selection. Give the rationale for the choice of cases and controls |  |  |  |
|  |  | Cross-sectional study—give the eligibility criteria, and the sources and methods of selection of participants |  |  |  |
|  |  | *(b)* Cohort study—for matched studies, give matching criteria and number of exposed and unexposed |  |  |  |
|  |  | Case-control study—for matched studies, give matching criteria and the number of controls per case |  |  |  |
| *Variables* | **7** | Clearly define all outcomes, exposures, predictors, potential confounders, and effect modifiers. Give diagnostic criteria, if applicable | X | 3–4 |  |
| *Data sources/ measurement* | **8*** | For each variable of interest, give sources of data and details of methods of assessment (measurement). Describe comparability of assessment methods if there is more than one group | X | 3–4 |  |
| *Bias* | **9** | Describe any efforts to address potential sources of bias | — | — | Information was not available |
| *Study size* | **10** | Explain how the study size was decided | — | — | Information was not available |
| *Quantitative variables* | **11** | Explain how quantitative variables were handled in the analyses. If applicable, describe which groupings were chosen and why | X | 3–4 |  |
| *Statistical methods* | **12** | *(a)* Describe all statistical methods, including those used to control for confounding | X | 4–5 |  |
|  |  | *(b)* Describe any methods used to examine subgroups and interactions | X | 4–5 |  |
|  |  | *(c)* Explain how missing data were addressed | — | — | Information was not available |
|  |  | *(d)* Cohort study—if applicable, explain how loss to follow-up was addressed | — | — | Information was not available |
|  |  | Case-control study—if applicable, explain how matching of cases and controls was addressed |  |  |  |
|  |  | Cross-sectional study—if applicable, describe analytical methods taking account of sampling strategy |  |  |  |
|  |  | *(e)* Describe any sensitivity analyses | X | 4–5 |  |
| **Results** |  |  |  |  |  |
| *Participants* | **13*** | *(a)* Report numbers of individuals at each stage of study—e.g., numbers potentially eligible, examined for eligibility, confirmed eligible, included in the study, completing follow-up, and analysed | — | — | Information was not available |
|  |  | *(b)* Give reasons for non-participation at each stage | — | — | Information was not available |
|  |  | *(c)* Consider use of a flow diagram | — | — | Information was not available |
| *Descriptive data* | **14*** | *(a)* Give characteristics of study participants (e.g., demographic, clinical, social) and information on exposures and potential confounders | — | — | Information was not available |
|  |  | *(b)* Indicate number of participants with missing data for each variable of interest | — | — | Information was not available |
|  |  | *(c)* Cohort study—summarise follow-up time (e.g., average and total amount) |  |  |  |
| *Outcome data* | **15*** | Cohort study—report numbers of outcome events or summary measures over time | X | Tables 1–3 |  |
|  |  | Case-control study—report numbers in each exposure category, or summary measures of exposure |  |  |  |
|  |  | Cross-sectional study—report numbers of outcome events or summary measures |  |  |  |
| *Main results* | **16** | *(a)* Give unadjusted estimates and, if applicable, confounder-adjusted estimates and their precision (e.g., 95% confidence interval). Make clear which confounders were adjusted for and why they were included | X | Table 3 |  |
|  |  | *(b)* Report category boundaries when continuous variables were categorized | X | Tables 1–2 |  |
|  |  | *(c)* If relevant, consider translating estimates of relative risk into absolute risk for a meaningful time period |  |  |  |
| *Other analyses* | **17** | Report other analyses done—e.g., analyses of subgroups and interactions, and sensitivity analyses | X | Table 3 |  |
| **Discussion** |  |  |  |  |  |
| *Key results* | **18** | Summarise key results with reference to study objectives | X | 6 (1 [1–8]) |  |
| *Limitations* | **19** | Discuss limitations of the study, taking into account sources of potential bias or imprecision. Discuss both direction and magnitude of any potential bias | X | 6, 7, 8 |  |
| *Interpretation* | **20** | Give a cautious overall interpretation of results considering objectives, limitations, multiplicity of analyses, results from similar studies, and other relevant evidence | X | 6, 7, 8 |  |
| *Generalisability* | **21** | Discuss the generalisability (external validity) of the study results | X | — | Researchers suggested several inferences about their data across the discussion section. |
| **Other information** |  |  |  |  |  |
| *Funding* | **22** | Give the source of funding and the role of the funders for the present study and, if applicable, for the original study on which the present article is based | X | Acknowledgments |  |
| **TOTAL SCORE:** 17 |  |  |  |  |  |

| **STROBE Statement—checklist of items that should be included in reports of observational studies** |  |  |  |  |  |
| --- | --- | --- | --- | --- | --- |
|  |  |  |  |  |  |
| **Article:** Dietary protein intake is associated with better physical function and muscle strength among elderly women | **Year:** 2016 |  |  |  |  |
| **Authors:**  Isanejad et al., |  |  |  |  |  |
|  |  |  |  |  |  |
|  | **Item No** | **Recommendation** | **Reported** | **Page (paragraph [line])** | **Note** |
| **Title and abstract** | **1** | *(a)* Indicate the study’s design with a commonly used term in the title or the abstract | X | 1281 |  |
|  |  | *(b)* Provide in the abstract an informative and balanced summary of what was done and what was found | X | 1281 |  |
| **Introduction** |  |  |  |  |  |
| *Background/rationale* | **2** | Explain the scientific background and rationale for the investigation being reported | X | 1281–1282 |  |
| *Objectives* | **3** | State specific objectives, including any prespecified hypotheses | X | 1282 (2 [4–10]) |  |
| **Methods** |  |  |  |  |  |
| *Study design* | **4** | Present key elements of study design early in the paper | X | 1282 (3 [1–20]) |  |
| *Setting* | **5** | Describe the setting, locations, and relevant dates, including periods of recruitment, exposure, follow-up, and data collection | X | 1282 (3 [1–20]) |  |
| *Participants* | **6** | *(a)* Cohort study—give the eligibility criteria, and the sources and methods of selection of participants. Describe methods of follow-up |  |  |  |
|  |  | Case-control study—give the eligibility criteria, and the sources and methods of case ascertainment and control selection. Give the rationale for the choice of cases and controls |  |  |  |
|  |  | Cross-sectional study—give the eligibility criteria, and the sources and methods of selection of participants | X | 1282 (3 [1–20]) |  |
|  |  | *(b)* Cohort study—for matched studies, give matching criteria and number of exposed and unexposed |  |  |  |
|  |  | Case-control study—for matched studies, give matching criteria and the number of controls per case |  |  |  |
| *Variables* | **7** | Clearly define all outcomes, exposures, predictors, potential confounders, and effect modifiers. Give diagnostic criteria, if applicable | X | 1282–1283 |  |
| *Data sources/ measurement* | **8*** | For each variable of interest, give sources of data and details of methods of assessment (measurement). Describe comparability of assessment methods if there is more than one group | X | 1282–1284 |  |
| *Bias* | **9** | Describe any efforts to address potential sources of bias | X | — | Researchers described that data acquisition were performed by trained researchers under rigorous standardized procedures. In addition, validated methods were used for evaluation. Finally, measurement errors were excluded. |
| *Study size* | **10** | Explain how the study size was decided | — | — | Information was not available |
| *Quantitative variables* | **11** | Explain how quantitative variables were handled in the analyses. If applicable, describe which groupings were chosen and why | X | 1282 (3 [1–6]), 1283 [1–20], (3 [1–9] |  |
| *Statistical methods* | **12** | *(a)* Describe all statistical methods, including those used to control for confounding | X | 1283 (3, 4, 5); 1384 [1–3] |  |
|  |  | *(b)* Describe any methods used to examine subgroups and interactions | X | 1283 (3, 4, 5); 1384 [1–3] |  |
|  |  | *(c)* Explain how missing data were addressed | — | — | Information was not available |
|  |  | *(d)* Cohort study—if applicable, explain how loss to follow-up was addressed |  |  |  |
|  |  | Case-control study—if applicable, explain how matching of cases and controls was addressed |  |  |  |
|  |  | Cross-sectional study—if applicable, describe analytical methods taking account of sampling strategy |  |  |  |
|  |  | *(e)* Describe any sensitivity analyses | X | 1283 (3, 4, 5); 1384 [1–3] |  |
| **Results** |  |  |  |  |  |
| *Participants* | **13*** | *(a)* Report numbers of individuals at each stage of study—e.g., numbers potentially eligible, examined for eligibility, confirmed eligible, included in the study, completing follow-up, and analysed | — | — | Information was not available |
|  |  | *(b)* Give reasons for non-participation at each stage | — | — | Information was not available |
|  |  | *(c)* Consider use of a flow diagram | — | — | Information was not available |
| *Descriptive data* | **14*** | *(a)* Give characteristics of study participants (e.g., demographic, clinical, social) and information on exposures and potential confounders | X | Table 1 |  |
|  |  | *(b)* Indicate number of participants with missing data for each variable of interest | — | — | Information was not available |
|  |  | *(c)* Cohort study—summarise follow-up time (e.g., average and total amount) |  |  |  |
| *Outcome data* | **15*** | Cohort study—report numbers of outcome events or summary measures over time |  |  |  |
|  |  | Case-control study—report numbers in each exposure category, or summary measures of exposure |  |  |  |
|  |  | Cross-sectional study—report numbers of outcome events or summary measures | X | Tables 1–5 |  |
| *Main results* | **16** | *(a)* Give unadjusted estimates and, if applicable, confounder-adjusted estimates and their precision (e.g., 95% confidence interval). Make clear which confounders were adjusted for and why they were included | X | Tables 3–5 |  |
|  |  | *(b)* Report category boundaries when continuous variables were categorized |  |  |  |
|  |  | *(c)* If relevant, consider translating estimates of relative risk into absolute risk for a meaningful time period |  |  |  |
| *Other analyses* | **17** | Report other analyses done—e.g., analyses of subgroups and interactions, and sensitivity analyses | X | Tables 3–5 |  |
| **Discussion** |  |  |  |  |  |
| *Key results* | **18** | Summarise key results with reference to study objectives | X | 1285 (3 [1–5]), 1286 (1 [1–21]) |  |
| *Limitations* | **19** | Discuss limitations of the study, taking into account sources of potential bias or imprecision. Discuss both direction and magnitude of any potential bias | X | 1285–1288 |  |
| *Interpretation* | **20** | Give a cautious overall interpretation of results considering objectives, limitations, multiplicity of analyses, results from similar studies, and other relevant evidence | X | 1285–1288 |  |
| *Generalisability* | **21** | Discuss the generalisability (external validity) of the study results | X | 1285–1288 |  |
| **Other information** |  |  |  |  |  |
| *Funding* | **22** | Give the source of funding and the role of the funders for the present study and, if applicable, for the original study on which the present article is based | X | Acknowledgments |  |
| **TOTAL SCORE: 20** |  |  |  |  |  |

| **STROBE Statement—checklist of items that should be included in reports of observational studies** |  |  |  |  |  |
| --- | --- | --- | --- | --- | --- |
|  |  |  |  |  |  |
| **Article:** Protein intake and distribution in relation to physical functioning and quality of life in community-dwelling elderly people: Acknowledging the role of physical activity | **Year:** 2018 |  |  |  |  |
| **Authors:** ten Haaf et al., |  |  |  |  |  |
|  |  |  |  |  |  |
|  | **Item No** | **Recommendation** | **Reported** | **Page (paragraph [line])** | **Note** |
| **Title and abstract** | **1** | *(a)* Indicate the study’s design with a commonly used term in the title or the abstract | X | 1 (3) |  |
|  |  | *(b)* Provide in the abstract an informative and balanced summary of what was done and what was found | X | 1 |  |
| **Introduction** |  |  |  |  |  |
| *Background/rationale* | **2** | Explain the scientific background and rationale for the investigation being reported | X | — |  |
| *Objectives* | **3** | State specific objectives, including any prespecified hypotheses | X | 2 (4[4–6]) |  |
| **Methods** |  |  |  |  |  |
| *Study design* | **4** | Present key elements of study design early in the paper | X | 2 (6 [1–5]) and 3 (1[1–2]) |  |
| *Setting* | **5** | Describe the setting, locations, and relevant dates, including periods of recruitment, exposure, follow-up, and data collection | — | — | Researchers describe from where the volunteers were recruited, but not the setting, relevant dates, as well as the periods of recruitment. |
| *Participants* | **6** | *(a)* Cohort study—give the eligibility criteria, and the sources and methods of selection of participants. Describe methods of follow-up | — | — | Researchers did not mention the eligibility criteria used in the present study. The methods of selection were poorly described, so that valuable information is not present. |
|  |  | Case-control study—give the eligibility criteria, and the sources and methods of case ascertainment and control selection. Give the rationale for the choice of cases and controls |  |  |  |
|  |  | Cross-sectional study—give the eligibility criteria, and the sources and methods of selection of participants |  |  |  |
|  |  | *(b)* Cohort study—for matched studies, give matching criteria and number of exposed and unexposed |  |  |  |
|  |  | Case-control study—for matched studies, give matching criteria and the number of controls per case |  |  |  |
| *Variables* | **7** | Clearly define all outcomes, exposures, predictors, potential confounders, and effect modifiers. Give diagnostic criteria, if applicable | X | 2 (6 [1–5]), 3 (1 [1–2]), 4 (5 [1–16]), and 5 (1 [1–2]). |  |
| *Data sources/ measurement* | **8*** | For each variable of interest, give sources of data and details of methods of assessment (measurement). Describe comparability of assessment methods if there is more than one group | X | 3 and 4 |  |
| *Bias* | **9** | Describe any efforts to address potential sources of bias | X | — | Researchers used validated methods for all measurements. In addition, trained researchers applied the tests. |
| *Study size* | **10** | Explain how the study size was decided | — | — | There was no description regarding the design of the study size |
| *Quantitative variables* | **11** | Explain how quantitative variables were handled in the analyses. If applicable, describe which groupings were chosen and why | X | 2 (6 [1–5]), 3, 4, 5 (1 [1–2] |  |
| *Statistical methods* | **12** | *(a)* Describe all statistical methods, including those used to control for confounding | X | 4 (5 [1–16]), and 5 (1 [1–2]). |  |
|  |  | *(b)* Describe any methods used to examine subgroups and interactions |  |  |  |
|  |  | *(c)* Explain how missing data were addressed |  |  |  |
|  |  | *(d)* Cohort study—if applicable, explain how loss to follow-up was addressed |  |  |  |
|  |  | Case-control study—if applicable, explain how matching of cases and controls was addressed |  |  |  |
|  |  | Cross-sectional study—if applicable, describe analytical methods taking account of sampling strategy |  |  |  |
|  |  | *(e)* Describe any sensitivity analyses |  |  |  |
| **Results** |  |  |  |  |  |
| *Participants* | **13*** | *(a)* Report numbers of individuals at each stage of study—e.g., numbers potentially eligible, examined for eligibility, confirmed eligible, included in the study, completing follow-up, and analysed | — | — | Although the researchers provided a wide range of information about the volunteers, there was no description regarding the report numbers of individuals at each stage of the study, as well as the reasons for non-participation. |
|  |  | *(b)* Give reasons for non-participation at each stage |  |  |  |
|  |  | *(c)* Consider use of a flow diagram |  |  |  |
| *Descriptive data* | **14*** | *(a)* Give characteristics of study participants (e.g., demographic, clinical, social) and information on exposures and potential confounders | X | Table 1 |  |
|  |  | *(b)* Indicate number of participants with missing data for each variable of interest | — | — | There was no description about missing data |
|  |  | *(c)* Cohort study—summarise follow-up time (e.g., average and total amount) |  |  |  |
| *Outcome data* | **15*** | Cohort study—report numbers of outcome events or summary measures over time |  |  |  |
|  |  | Case-control study—report numbers in each exposure category, or summary measures of exposure |  |  |  |
|  |  | Cross-sectional study—report numbers of outcome events or summary measures | X | Tables 2 and Figures 1, 2, 3 and 4. |  |
| *Main results* | **16** | *(a)* Give unadjusted estimates and, if applicable, confounder-adjusted estimates and their precision (e.g., 95% confidence interval). Make clear which confounders were adjusted for and why they were included | X | Tables 2 and Figures 1, 2, 3 and 4. | Results section made clear the process of analysis, including the adjust for confounders |
|  |  | *(b)* Report category boundaries when continuous variables were categorized |  |  |  |
|  |  | *(c)* If relevant, consider translating estimates of relative risk into absolute risk for a meaningful time period |  |  |  |
| *Other analyses* | **17** | Report other analyses done—e.g., analyses of subgroups and interactions, and sensitivity analyses | X | Figures 1, 2, 3 and 4 | Researchers performed a wide range of subgroup analysis considering protein intake distribution pattern and physical function |
| **Discussion** |  |  |  |  |  |
| *Key results* | **18** | Summarise key results with reference to study objectives | X | 9 (1 [1–6]) |  |
| *Limitations* | **19** | Discuss limitations of the study, taking into account sources of potential bias or imprecision. Discuss both direction and magnitude of any potential bias | X | 10 (2 [1–11]) |  |
| *Interpretation* | **20** | Give a cautious overall interpretation of results considering objectives, limitations, multiplicity of analyses, results from similar studies, and other relevant evidence | X | 9 and 10 | Researchers mentioned other relevant evidence across all discussion section. The discussion is not biased, and is a clear discussion about the findings may be observed. |
| *Generalisability* | **21** | Discuss the generalisability (external validity) of the study results | X | 9 (2 [1–15]) and 10 (1 [6–7]) |  |
| **Other information** |  |  |  |  |  |
| *Funding* | **22** | Give the source of funding and the role of the funders for the present study and, if applicable, for the original study on which the present article is based | X | Acknowledgments |  |
| **TOTAL SCORE: 19** |  |  |  |  |  |

| **Table S2. Individual quality assessment analysis of each included study** | | | | | |
| --- | --- | --- | --- | --- | --- |
| **STROBE Statement—checklist of items that should be included in reports of observational studies** | | | | | |
|  |  |  |  |  |  |
| **Article:** Adequate dietary protein is associated with better physical performance among post-menopausal women 60–90 years. | **Year:** 2014 |  |  |  |  |
| **Authors:**  Gregorio et al., |  |  |  |  |  |
|  |  |  |  |  |  |
|  | **Item No** | **Recommendation** | **Reported** | **Page (paragraph [line])** | **Note** |
| **Title and abstract** | **1** | *(a)* Indicate the study’s design with a commonly used term in the title or the abstract |  |  |  |
|  |  | *(b)* Provide in the abstract an informative and balanced summary of what was done and what was found | X | 1729 |  |
| **Introduction** |  |  |  |  |  |
| *Background/rationale* | **2** | Explain the scientific background and rationale for the investigation being reported | X | 1729–1730 |  |
| *Objectives* | **3** | State specific objectives, including any prespecified hypotheses | X | 1730 |  |
| **Methods** |  |  |  |  |  |
| *Study design* | **4** | Present key elements of study design early in the paper | X | 1730 |  |
| *Setting* | **5** | Describe the setting, locations, and relevant dates, including periods of recruitment, exposure, follow-up, and data collection | X | 1730–1731 |  |
| *Participants* | **6** | *(a)* Cohort study—give the eligibility criteria, and the sources and methods of selection of participants. Describe methods of follow-up | X | 1730 |  |
|  |  | Case-control study—give the eligibility criteria, and the sources and methods of case ascertainment and control selection. Give the rationale for the choice of cases and controls |  |  |  |
|  |  | Cross-sectional study—give the eligibility criteria, and the sources and methods of selection of participants |  |  |  |
|  |  | *(b)* Cohort study—for matched studies, give matching criteria and number of exposed and unexposed |  |  |  |
|  |  | Case-control study—for matched studies, give matching criteria and the number of controls per case |  |  |  |
| *Variables* | **7** | Clearly define all outcomes, exposures, predictors, potential confounders, and effect modifiers. Give diagnostic criteria, if applicable | X | 1730–1731 |  |
| *Data sources/ measurement* | **8*** | For each variable of interest, give sources of data and details of methods of assessment (measurement). Describe comparability of assessment methods if there is more than one group | X | 1731 |  |
| *Bias* | **9** | Describe any efforts to address potential sources of bias | — | Information was not available |  |
| *Study size* | **10** | Explain how the study size was decided | X |  |  |
| *Quantitative variables* | **11** | Explain how quantitative variables were handled in the analyses. If applicable, describe which groupings were chosen and why | X |  |  |
| *Statistical methods* | **12** | *(a)* Describe all statistical methods, including those used to control for confounding |  |  |  |
|  |  | *(b)* Describe any methods used to examine subgroups and interactions |  |  |  |
|  |  | *(c)* Explain how missing data were addressed |  |  | 1730–1732 |
|  |  | *(d)* Cohort study—if applicable, explain how loss to follow-up was addressed |  |  |  |
|  |  | Case-control study—if applicable, explain how matching of cases and controls was addressed |  | X |  |
|  |  | Cross-sectional study—if applicable, describe analytical methods taking account of sampling strategy |  |  |  |
|  |  | *(e)* Describe any sensitivity analyses |  |  |  |
| **Results** |  |  |  |  |  |
| *Participants* | **13*** | *(a)* Report numbers of individuals at each stage of study—e.g., numbers potentially eligible, examined for eligibility, confirmed eligible, included in the study, completing follow-up, and analysed | X |  | 1732 |
|  |  | *(b)* Give reasons for non-participation at each stage |  |  |  |
|  |  | *(c)* Consider use of a flow diagram |  |  |  |
| *Descriptive data* | **14*** | *(a)* Give characteristics of study participants (e.g., demographic, clinical, social) and information on exposures and potential confounders |  |  |  |
|  |  | *(b)* Indicate number of participants with missing data for each variable of interest | X |  | Table 1; Individuals with missing data were excluded |
|  |  | *(c)* Cohort study—summarise follow-up time (e.g., average and total amount) |  |  |  |
| *Outcome data* | **15*** | Cohort study—report numbers of outcome events or summary measures over time |  |  |  |
|  |  | Case-control study—report numbers in each exposure category, or summary measures of exposure |  |  |  |
|  |  | Cross-sectional study—report numbers of outcome events or summary measures | X |  |  |
| *Main results* | **16** | *(a)* Give unadjusted estimates and, if applicable, confounder-adjusted estimates and their precision (e.g., 95% confidence interval). Make clear which confounders were adjusted for and why they were included |  |  |  |
|  |  | *(b)* Report category boundaries when continuous variables were categorized |  |  |  |
|  |  | *(c)* If relevant, consider translating estimates of relative risk into absolute risk for a meaningful time period | X |  |  |
| *Other analyses* | **17** | Report other analyses done—e.g., analyses of subgroups and interactions, and sensitivity analyses | — |  |  |
| **Discussion** |  |  |  |  |  |
| *Key results* | **18** | Summarise key results with reference to study objectives | X |  |  |
| *Limitations* | **19** | Discuss limitations of the study, taking into account sources of potential bias or imprecision. Discuss both direction and magnitude of any potential bias | X |  |  |
| *Interpretation* | **20** | Give a cautious overall interpretation of results considering objectives, limitations, multiplicity of analyses, results from similar studies, and other relevant evidence | X |  |  |
| *Generalisability* | **21** | Discuss the generalisability (external validity) of the study results | X |  |  |
| **Other information** |  |  |  |  |  |
| *Funding* | **22** | Give the source of funding and the role of the funders for the present study and, if applicable, for the original study on which the present article is based | X |  |  |
| **TOTAL SCORE:** 20 |  |  |  |  |  |
